# Supplementary material for: On the variability of dynamic functional connectivity assessment methods
Source: Gigascience. 2024 Apr 8;13:giae009. doi: 10.1093/gigascience/giae009 (PMC11000510; doi:10.1093/gigascience/giae009)
Supplement: giae009_Supplemental_Files [file giae009_supplemental_files.zip › Supplementary Files/SuppTables/Supp_Tables_Bibliography.pdf]

References

[1] Mohammed Isam Al-Hiyali, Norashikin Yahya, Ibrahima Faye, and Ahmed Faeq Hussein. Identification of Autism Subtypes Based on Wavelet Coherence of BOLD FMRI Signals Using Convolutional Neural Network. *Sensors (Basel, Switzerland)*, 21(16):5256, August 2021.

[2] Antoine Bernas, Albert P. Aldenkamp, and Svitlana Zinger. Wavelet coherence-based classifier: A resting-state functional MRI study on neurodynamics in adolescents with high-functioning autism. *Computer Methods and Programs in Biomedicine*, 154:143–151, February 2018.

[3] N. de Lacy, D. Doherty, B. H. King, S. Rachakonda, and V. D. Calhoun. Disruption to control network function correlates with altered dynamic connectivity in the wider autism spectrum. *NeuroImage: Clinical*, 15:513–524, January 2017.

[4] Pingting Lin, Shiyi Zang, Yi Bai, and Haixian Wang. Reconfiguration of Brain Network Dynamics in Autism Spectrum Disorder Based on Hidden Markov Model. *Frontiers in Human Neuroscience*, 16:774921, 2022.

[5] Jianping Qiao, Rong Wang, Hongjia Liu, Guangrun Xu, and Zhishun Wang. Brain disorder prediction with dynamic multivariate spatio-temporal features: Application to Alzheimer's disease and autism spectrum disorder. *Frontiers in Aging Neuroscience*, 14, 2022.

[6] Sophie Dautricourt, Julie Gonneaud, Brigitte Landeau, Vince D. Calhoun, Robin de Flores, Géraldine Poisnel, Salma Bougacha, Valentin Ourry, Edelweiss Touron, Elizabeth Kuhn, Harriet Demintz-King, Natalie L. Marchant, Denis Vivien, Vincent de la Sayette, Antoine Lutz, Gaël Chételat, Eider M. Arenaza-Urquijo, Florence Allais, Claire André, Julien Asselineau, Alexandre Bejanin, Pierre Champetier, Gaël Chételat, Anne Chocat, Sophie Dautricourt, Robin de Flores, Marion Delarue, Stéphanie Egret, Francesca Felisatti, Eglantine Ferrand Devouge, Eric Frison, Julie Gonneaud, Marc Heidmann, Thien Huong Tran, Elizabeth Kuhn, Gwendoline le Du, Brigitte Landeau, Valérie Lefranc, Antoine Lutz, Florence Mezenge, Inès Moulinet, Valentin Ourry, Cassandre Palix, Léo Paly, Géraldine Poisnel, Anne Quillard, Géraldine Rauchs, Stéphane Rehel, Florence Requier, Edelweiss Touron, Denis Vivien, Caitlin Ware, Sebastian Baez Lugo, Olga Klimecki, Patrik Vuilleumier, Thorsten Barnhofer, Fabienne Collette, Eric Salmon, Vincent de la Sayette, Pascal Delamillieure, Martine Batchelor, Axel Beaugonin, Francis Gheysen, Harriet Demnitz-King, Natalie Marchant, Tim Whitfield, Corinne Schimmer, Miranka Wirth, and for the Medit-Ageing Research Group. Dynamic functional connectivity patterns associated with dementia risk. *Alzheimer's Research & Therapy*, 14(1):72, May 2022.

[7] David T. Jones, Prashanthi Vemuri, Matthew C. Murphy, Jeffrey L. Gunter, Matthew L. Senjem, Mary M. Machulda, Scott A. Przybelski, Brian E. Gregg, Kejal Kantarci, David S. Knopman, Bradley F. Boeve, Ronald C. Petersen, and Clifford R. Jack Jr. Non-Stationarity in the "Resting Brain's" Modular Architecture. *PLOS ONE*, 7(6):e39731, June 2012. Publisher: Public Library of Science.

[8] Mohammad S. E. Sendi, Elaheh Zendehrouh, Robyn L. Miller, Zening Fu, Yuhui Du, Jingyu Liu, Elizabeth C. Mormino, David H. Salat, and Vince D. Calhoun. Alzheimer's Disease Projection From Normal to Mild Dementia Reflected in Functional Network Connectivity: A Longitudinal Study. *Frontiers in Neural Circuits*, 14:593263, 2020.

[9] Aldo Córdova-Palomera, Tobias Kaufmann, Karin Persson, Dag Alnæs, Nhat Trung Doan, Torgeir Moberget, Martina Jonette Lund, Maria Lage Barca, Andreas Engvig, Anne Brækhus, Knut Engedal, Ole A. Andreassen, Geir Selbæk, and Lars T. Westlye. Disrupted global metastability and static and dynamic brain connectivity across individuals in the Alzheimer's disease continuum. *Scientific Reports*, 7(1):40268, January 2017. Number: 1 Publisher: Nature Publishing Group.

[10] Heung-Il Suk, Chong-Yaw Wee, Seong-Whan Lee, and Dinggang Shen. State-space model with deep learning for functional dynamics estimation in resting-state fMRI. *NeuroImage*, 129:292–307, April 2016.

[11] Biao Jie, Mingxia Liu, and Dinggang Shen. Integration of temporal and spatial properties of dynamic connectivity networks for automatic diagnosis of brain disease. *Medical Image Analysis*, 47:81–94, July 2018.

[12] Chong-Yaw Wee, Sen Yang, Pew-Thian Yap, and Dinggang Shen. Sparse Temporally Dynamic Resting-State Functional Connectivity Networks for Early MCI Identification. *Brain imaging and behavior*, 10(2):342–356, June 2016.

[13] Xiaobo Chen, Han Zhang, Lichi Zhang, Celina Shen, Seong-Whan Lee, and Dinggang Shen. Extraction of dynamic functional connectivity from brain grey matter and white matter for MCI classification. *Human Brain Mapping*, 38(10):5019–5034, 2017. _eprint: https://onlinelibrary.wiley.com/doi/pdf/10.1002/hbm.23711.

[14] Maziar Yaesoubi, Robyn L. Miller, Juan Bustillo, Kelvin O. Lim, Jatin Vaidya, and Vince D. Calhoun. A joint time-frequency analysis of resting-state functional connectivity reveals novel patterns of connectivity shared between or unique to schizophrenia patients and healthy controls. *NeuroImage: Clinical*, 15:761–768, January 2017.

[15] E. Damaraju, E. A. Allen, A. Belger, J. M. Ford, S. McEwen, D. H. Mathalon, B. A. Mueller, G. D. Pearlson, S. G. Potkin, A. Preda, J. A. Turner, J. G. Vaidya, T. G. van Erp, and V. D. Calhoun. Dynamic functional connectivity analysis reveals transient states of dysconnectivity in schizophrenia. *NeuroImage: Clinical*, 5:298–308, January 2014.

[16] Yuhui Du, Susanna L. Fryer, Zening Fu, Dongdong Lin, Jing Sui, Jiayu Chen, Eswar Damaraju, Eva Mennigen, Barbara Stuart, Rachel L. Loewy, Daniel H. Mathalon, and Vince D. Calhoun. Dynamic functional connectivity impairments in early schizophrenia and clinical high-risk for psychosis. *NeuroImage*, 180:632–645, October 2018.

[17] Mohammad S. E. Sendi, Elaheh Zendehrouh, Jessica A. Turner, and Vince D. Calhoun. Dynamic patterns within the default mode network in

schizophrenia subgroups. *Annual International Conference of the IEEE Engineering in Medicine and Biology Society. IEEE Engineering in Medicine and Biology Society. Annual International Conference*, 2021:1640–1643, November 2021.

[18] Akhil Kottaram, Leigh A. Johnston, Luca Cocchi, Eleni P. Ganella, Ian Everall, Christos Pantelis, Ramamohanarao Kotagiri, and Andrew Zalesky. Brain network dynamics in schizophrenia: Reduced dynamism of the default mode network. *Human Brain Mapping*, 40(7):2212–2228, 2019. _eprint: https://onlinelibrary.wiley.com/doi/pdf/10.1002/hbm.24519.

[19] Barnaly Rashid, Mohammad R. Arbabshirani, Eswar Damaraju, Mustafa S. Cetin, Robyn Miller, Godfrey D. Pearlson, and Vince D. Calhoun. Classification of schizophrenia and bipolar patients using static and dynamic resting-state fMRI brain connectivity. *NeuroImage*, 134:645–657, July 2016.

[20] María Díez-Cirarda, Antonio P. Strafella, Jinhee Kim, Javier Peña, Natalia Ojeda, Alberto Cabrera-Zubizarreta, and Naroa Ibarretxe-Bilbao. Dynamic functional connectivity in Parkinson's disease patients with mild cognitive impairment and normal cognition. *NeuroImage: Clinical*, 17:847–855, January 2018.

[21] Gwenda Engels, Annemarie Vlaar, Brónagh McCoy, Erik Scherder, and Linda Douw. Dynamic Functional Connectivity and Symptoms of Parkinson's Disease: A Resting-State fMRI Study. *Frontiers in Aging Neuroscience*, 10:388, November 2018.

[22] Jinhee Kim, Marion Criaud, Sang Soo Cho, María Díez-Cirarda, Alexander Mihaescu, Sarah Coakeley, Christine Ghadery, Mikaeel Valli, Mark F Jacobs, Sylvain Houle, and Antonio P Strafella. Abnormal intrinsic brain functional network dynamics in Parkinson's disease. *Brain*, 140(11):2955–2967, November 2017.

[23] Dietmar Cordes, Xiaowei Zhuang, Muhammad Kaleem, Karthik Sreenivasan, Zhengshi Yang, Virendra Mishra, Sarah J. Banks, Brent Bluett, and Jeffrey L. Cummings. Advances in functional magnetic resonance imaging data analysis methods using Empirical Mode Decomposition to investigate temporal changes in early Parkinson's disease. *Alzheimer's & Dementia: Translational Research & Clinical Interventions*, 4(1):372–386, 2018. _eprint: https://onlinelibrary.wiley.com/doi/pdf/10.1016/j.trci.2018.04.009.

[24] Aiping Liu, Sue-Jin Lin, Taomian Mi, Xun Chen, Piu Chan, Z. Jane Wang, and Martin J. McKeown. Decreased subregional specificity of the putamen in Parkinson's Disease revealed by dynamic connectivity-derived parcellation. *NeuroImage: Clinical*, 20:1163–1175, January 2018.

[25] MadhyasthaTara M, AskrenMary K, BoordPeter, and GrabowskiThomas J. Dynamic Connectivity at Rest Predicts Attention Task Performance. *Brain Connectivity*, February 2015. Publisher: Mary Ann Liebert, Inc. 140 Huguenot Street, 3rd Floor New Rochelle, NY 10801 USA.

[26] Xiaowei Zhuang, Ryan R. Walsh, Karthik Sreenivasan, Zhengshi Yang, Virendra Mishra, and Dietmar Cordes. Incorporating spatial constraint in co-activation pattern analysis to explore the dynamics of resting-state networks: An application to Parkinson's disease. *NeuroImage*, 172:64–84, May 2018.

[27] Jinli Ou, Li Xie, Changfeng Jin, Xiang Li, Dajiang Zhu, Rongxin Jiang, Yaowu Chen, Jing Zhang, Lingjiang Li, and Tianming Liu. Characterizing and Differentiating Brain State Dynamics via Hidden Markov Models. *Brain Topography*, 28(5):666–679, September 2015.

[28] Marina Charquero-Ballester, Birgit Kleim, Diego Vidaurre, Christian Ruff, Eloise Stark, Jetro J. Tuulari, Hugh McManners, Yair Bar-Haim, Linda Bouquillon, Allison Moseley, Steven C. R. Williams, Mark W. Woolrich, Morten L. Kringelbach, and Anke Ehlers. Effective psychological therapy for PTSD changes the dynamics of specific large-scale brain networks. *Human Brain Mapping*, 43(10):3207–3220, 2022. _eprint: https://onlinelibrary.wiley.com/doi/pdf/10.1002/hbm.25846.

[29] Xueling Suo, Chao Zuo, Huan Lan, Wenbin Li, Lingjiang Li, Graham J. Kemp, Song Wang, and Qiyong Gong. Multilayer Network Analysis of Dynamic Network Reconfiguration in Adults with Posttraumatic Stress Disorder. *Biological Psychiatry: Cognitive Neuroscience and Neuroimaging*, September 2022.

[30] Changfeng Jin, Hao Jia, Pradyumna Lanka, D Rangaprakash, Lingjiang Li, Tianming Liu, Xiaoping Hu, and Gopikrishna Deshpande. Dynamic brain connectivity is a better predictor of PTSD than static connectivity. *Human Brain Mapping*, 38(9):4479–4496, 2017. _eprint: https://onlinelibrary.wiley.com/doi/pdf/10.1002/hbm.23676.

[31] Elaheh Zendehrouh, Mohammad. S. E. Sendi, Jing Sui, Zening Fu, Dongmei Zhi, Luxian Lv, Xiaohong Ma, Qing Ke, Xianbin Li, Chuanyue Wang, Christopher. C. Abbott, Jessica A. Turner, Robyn. L Miller, and Vince D. Calhoun. Aberrant Functional Network Connectivity Transition Probability in Major Depressive Disorder. *Annual International Conference of the IEEE Engineering in Medicine and Biology Society. IEEE Engineering in Medicine and Biology Society. Annual International Conference*, 2020:1493–1496, July 2020.

[32] Roselinde H. Kaiser, Susan Whitfield-Gabrieli, Daniel G. Dillon, Franziska Goer, Miranda Beltzer, Jared Minkel, Moria Smoski, Gabriel Dichter, and Diego A. Pizzagalli. Dynamic Resting-State Functional Connectivity in Major Depression. *Neuropsychopharmacology*, 41(7):1822–1830, June 2016. Bandiera_abtest: a Cg_type: Nature Research Journals Number: 7 Primary_atype: Research Publisher: Nature Publishing Group Subject_term: Depression Subject_term_id: depression.

[33] Dongmei Zhi, Vince D. Calhoun, Luxian Lv, Xiaohong Ma, Qing Ke, Zening Fu, Yuhui Du, Yongfeng Yang, Xiao Yang, Miao Pan, Shile Qi, Rongtao Jiang, Qingbao Yu, and Jing Sui. Aberrant Dynamic Functional Network Connectivity and Graph Properties in Major Depressive Disorder. *Frontiers in Psychiatry*, 9, 2018.

[34] Nina de Lacy and Vince D. Calhoun. Dynamic connectivity and the effects of maturation in youth with attention deficit hyperactivity disorder. *Network Neuroscience*, 3(1):195–216, December 2018.

[35] Marion Sourty, Laurent Thoraval, Daniel Roquet, Jean-Paul Armspach, Jack Foucher, and Frédéric Blanc. Identifying Dynamic Functional Connectivity Changes in Dementia with Lewy Bodies Based on Product Hidden Markov Models. *Frontiers in Computational Neuroscience*, 10:60, 2016.

[36] Feng Liu, Yifeng Wang, Meiling Li, Wenqin Wang, Rong Li, Zhiqiang Zhang, Guangming Lu, and Huafu Chen. Dynamic functional network connectivity in idiopathic generalized epilepsy with generalized tonic–clonic seizure. *Human Brain Mapping*, 38(2):957–973, 2017. _eprint: https://onlinelibrary.wiley.com/doi/pdf/10.1002/hbm.23430.

[37] Chiara Favaretto, Michele Allegra, Gustavo Deco, Nicholas V. Metcalf, Joseph C. Griffis, Gordon L. Shulman, Andrea Brovelli, and Maurizio Corbetta. Subcortical-cortical dynamical states of the human brain and their breakdown in stroke. *Nature Communications*, 13(1):5069, August 2022. Number: 1 Publisher: Nature Publishing Group.

[38] Enrico Premi, Vince D. Calhoun, Matteo Diano, Stefano Gazzina, Maura Cosseddu, Antonella Alberici, Silvana Archetti, Donata Paternicò, Roberto Gasparotti, John van Swieten, Daniela Galimberti, Raquel Sanchez-Valle, Robert Laforce, Fermin Moreno, Matthis Synofzik, Caroline Graff, Mario Masellis, Maria Carmela Tartaglia, James Rowe, Rik Vandenberghe, Elizabeth Finger, Fabrizio Tagliavini, Alexandre de Mendonça, Isabel Santana, Chris Butler, Simon Ducharme, Alex Gerhard, Adrian Danek, Johannes Levin, Markus Otto, Giovanni Frisoni, Stefano Cappa, Sandro Sorbi, Alessandro Padovani, Jonathan D. Rohrer, Barbara Borroni, Maria Rosario Almeida, Sarah Anderl-Straub, Christin Andersson, Anna Antonell, Andrea Arighi, Mircea Balasa, Myriam Barandiaran, Nuria Bargalló, Robart Bartha, Benjamin Bender, Luisa Benussi, Giuliano Binetti, Sandra Black, Martina Bocchetta, Sergi Borrego-Ecija, Jose Bras, Rose Bruffaerts, Paola Caroppo, David Cash, Miguel Castelo-Branco, Rhian Convery, Thomas Cope, María de Arriba, Giuseppe Di Fede, Zigor Díaz, Katrina M. Dick, Diana Duro, Chiara Fenoglio, Carlos Ferreira, Catarina B. Ferreira, Toby Flanagan, Nick Fox, Morris Freedman, Giorgio Fumagalli, Alazne Gabilondo, Serge Gauthier, Roberta Ghidoni, Giorgio Giaccone, Ana Gorostidi, Caroline Greaves, Rita Guerreiro, Carolin Heller, Tobias Hoegen, Begoña Indakoetxea, Vesna Jelic, Lize Jiskoot, Hans-Otto Karnath, Ron Keren, Maria João Leitão, Albert Lladó, Gemma Lombardi, Sandra Loosli, Carolina Maruta, Simon Mead, Lieke Meeter, Gabriel Miltenberger, Rick van Minkelen, Sara Mitchell, Benedetta Nacmias, Mollie Neason, Jennifer Nicholas, Linn Öijerstedt, Jaume Olives, Jessica Panman, Janne Papma, Maximilian Patzig, Michela Pievani, Sara Prioni, Catharina Prix, Rosa Rademakers, Veronica Redaelli, Tim Rittman, Ekaterina Rogaeva, Pedro Rosa-Neto, Giacomina Rossi, Martin Rossor, Beatriz Santiago, Elio Scarpini, Elisa Semler, Rachelle Shafei, Christen Shoesmith, Miguel Tábuas-Pereira, Mikel Tainta, David Tang-Wai, David L. Thomas, Hakan Thonberg, Carolyn Timberlake, Pietro Tiraboschi, Philip Vandamme, Mathieu Vandenbulcke, Michele Veldsman, Ana Verdelho, Jorge

Villanua, Jason Warren, Carlo Wilke, Henrik Zetterberg, and Miren Zulaica. The inner fluctuations of the brain in presymptomatic Frontotemporal Dementia: The chronnectome fingerprint. *NeuroImage*, 189:645–654, April 2019.

[39] Bolin Cao, Yan Chen, Ronghao Yu, Lixiang Chen, Ping Chen, Yihe Weng, Qinyuan Chen, Jie Song, Qiuyou Xie, and Ruiwang Huang. Abnormal dynamic properties of functional connectivity in disorders of consciousness. *NeuroImage: Clinical*, 24:102071, January 2019.

[40] Yuanyuan Chen, Weiwei Wang, Xin Zhao, Miao Sha, Ya'nan Liu, Xiong Zhang, Jianguo Ma, Hongyan Ni, and Dong Ming. Age-Related Decline in the Variation of Dynamic Functional Connectivity: A Resting State Analysis. *Frontiers in Aging Neuroscience*, 9, 2017.

[41] Zewei Wang, Qing Yang, and Li Min Chen. Abnormal dynamics of cortical resting state functional connectivity in chronic headache patients. *Magnetic Resonance Imaging*, 36:56–67, February 2017.

[42] Linda Douw, Mirjam Quaak, Sophie M. D. D. Fitzsimmons, Stella J. de Wit, Ysbrand D. van der Werf, Odile A. van den Heuvel, and Chris Vriend. Static and dynamic network properties of the repetitive transcranial magnetic stimulation target predict changes in emotion regulation in obsessive-compulsive disorder. *Brain Stimulation*, 13(2):318–326, March 2020.

[43] Majd Abdallah, Natalie M. Zahr, Manojkumar Saranathan, Nicolas Honnorat, Nicolas Farrugia, Adolf Pfefferbaum, Edith V. Sullivan, and Sandra Chanraud. Altered Cerebro-Cerebellar Dynamic Functional Connectivity in Alcohol Use Disorder: a Resting-State fMRI Study. *Cerebellum (London, England)*, 20(6):823–835, December 2021.

[44] Jie Zhang, Wei Cheng, Zhaowen Liu, Kai Zhang, Xu Lei, Ye Yao, Benjamin Becker, Yicen Liu, Keith M. Kendrick, Guangming Lu, and Jianfeng Feng. Neural, electrophysiological and anatomical basis of brain-network variability and its characteristic changes in mental disorders. *Brain*, 139(8):2307–2321, August 2016.

[45] Urs Braun, Axel Schäfer, Henrik Walter, Susanne Erk, Nina Romanczuk-Seiferth, Leila Haddad, Janina I. Schweiger, Oliver Grimm, Andreas Heinz, Heike Tost, Andreas Meyer-Lindenberg, and Danielle S. Bassett. Dynamic reconfiguration of frontal brain networks during executive cognition in humans. *Proceedings of the National Academy of Sciences*, 112(37):11678–11683, September 2015.

[46] Jingyuan E. Chen, Catie Chang, Michael D. Greicius, and Gary H. Glover. Introducing co-activation pattern metrics to quantify spontaneous brain network dynamics. *NeuroImage*, 111:476–488, May 2015.

[47] Julian Gaviria, Gwladys Rey, Thomas Bolton, Jaime Delgado, Dimitri Van De Ville, and Patrik Vuilleumier. Brain functional connectivity dynamics at rest in the aftermath of affective and cognitive challenges. *Human Brain Mapping*, 42(4):1054–1069, March 2021.

[48] Hua Xie, Charles Y. Zheng, Daniel A. Handwerker, Peter A. Bandettini, Vince D. Calhoun, Sunanda Mitra, and Javier Gonzalez-Castillo. Efficacy of different dynamic functional connectivity methods to capture cognitively relevant information. *NeuroImage*, 188:502–514, March 2019.

[49] Javier Gonzalez-Castillo, Colin W. Hoy, Daniel A. Handwerker, Meghan E. Robinson, Laura C. Buchanan, Ziad S. Saad, and Peter A. Bandettini. Tracking ongoing cognition in individuals using brief, whole-brain functional connectivity patterns. *Proceedings of the National Academy of Sciences*, 112(28):8762–8767, July 2015. Publisher: Proceedings of the National Academy of Sciences.

[50] Lucina Q. Uddin. Cognitive and behavioural flexibility: neural mechanisms and clinical considerations. *Nature Reviews Neuroscience*, 22(3):167–179, March 2021. Number: 3 Publisher: Nature Publishing Group.

[51] Chenhao Tan, Xin Liu, and Gaoyan Zhang. Inferring Brain State Dynamics Underlying Naturalistic Stimuli Evoked Emotion Changes With dHA-HMM. *Neuroinformatics*, 20(3):737–753, July 2022.

[52] A. B. A. Stevner, D. Vidaurre, J. Cabral, K. Rapuano, S. F. V. Nielsen, E. Tagliazucchi, H. Laufs, P. Vuust, G. Deco, M. W. Woolrich, E. Van Someren, and M. L. Kringelbach. Discovery of key whole-brain transitions and dynamics during human wakefulness and non-REM sleep. *Nature Communications*, 10(1):1035, March 2019. Number: 1 Publisher: Nature Publishing Group.

[53] Majd Abdallah, Nicolas Farrugia, Valentine Chirokoff, and Sandra Chanraud. Static and dynamic aspects of cerebro-cerebellar functional connectivity are associated with self-reported measures of impulsivity: A resting-state fMRI study. *Network Neuroscience*, 4(3):891–909, September 2020.

[54] Meiling Li, Louisa Dahmani, Danhong Wang, Jianxun Ren, Sophia Stocklein, Yuanxiang Lin, Guoming Luan, Zhiqiang Zhang, Guangming Lu, Fanziska Galiè, Ying Han, Alvaro Pascual-Leone, Meiyun Wang, Michael D. Fox, and Hesheng Liu. Co-activation patterns across multiple tasks reveal robust anti-correlated functional networks. *NeuroImage*, 227:117680, February 2021.

[55] Lorena G. A. Freitas, Thomas A. W. Bolton, Benjamin E. Krikler, Delphine Jochaut, Anne-Lise Giraud, Petra S. Hüppi, and Dimitri Van De Ville. Time-resolved effective connectivity in task fMRI: Psychophysiological interactions of Co-Activation patterns. *NeuroImage*, 212:116635, May 2020.

[56] Xiaolu Kong, Ru Kong, Csaba Orban, Peng Wang, Shaoshi Zhang, Kevin Anderson, Avram Holmes, John D. Murray, Gustavo Deco, Martijn van den Heuvel, and B. T. Thomas Yeo. Sensory-motor cortices shape functional connectivity dynamics in the human brain. *Nature Communications*, 12(1):6373, November 2021. Number: 1 Publisher: Nature Publishing Group.

[57] Barnaly Rashid, Jiayu Chen, Ishtiaque Rashid, Eswar Damaraju, Jingyu Liu, Robyn Miller, Oktay Agcaoglu, Theo G. M. van Erp, Kelvin O. Lim,

Jessica A. Turner, Daniel H. Mathalon, Judith M. Ford, James Voyvodic, Bryon A. Mueller, Aysenil Belger, Sarah McEwen, Steven G. Potkin, Adrian Preda, Juan R. Bustillo, Godfrey D. Pearlson, and Vince D. Calhoun. A framework for linking resting-state chronnectome/genome features in schizophrenia: A pilot study. *NeuroImage*, 184:843–854, January 2019.

[58] E. A. Allen, E. Damaraju, T. Eichele, L. Wu, and V. D. Calhoun. EEG Signatures of Dynamic Functional Network Connectivity States. *Brain Topography*, 31(1):101–116, January 2018.

[59] Gemeng Zhang, Biao Cai, Aiying Zhang, Julia M. Stephen, Tony W. Wilson, Vince D. Calhoun, and Yu-Ping Wang. Estimating Dynamic Functional Brain Connectivity With a Sparse Hidden Markov Model. *IEEE transactions on medical imaging*, 39(2):488–498, February 2020.

[60] Anees Abrol, Eswar Damaraju, Robyn L. Miller, Julia M. Stephen, Eric D. Claus, Andrew R. Mayer, and Vince D. Calhoun. Replicability of time-varying connectivity patterns in large resting state fMRI samples. *NeuroImage*, 163:160–176, December 2017.

[61] Huaze Xu, Hui Shen, Lubin Wang, Qi Zhong, Yu Lei, Liu Yang, Ling-Li Zeng, Zongtan Zhou, Dewen Hu, and Zheng Yang. Impact of 36h of total sleep deprivation on resting-state dynamic functional connectivity. *Brain Research*, 1688:22–32, June 2018.

[62] Timothy O. Laumann, Abraham Z. Snyder, Anish Mitra, Evan M. Gordon, Caterina Gratton, Babatunde Adeyemo, Adrian W. Gilmore, Steven M. Nelson, Jeff J. Berg, Deanna J. Greene, John E. McCarthy, Enzo Tagliazucchi, Helmut Laufs, Bradley L. Schlaggar, Nico U. F. Dosenbach, and Steven E. Petersen. On the Stability of BOLD fMRI Correlations. *Cerebral Cortex*, 27(10):4719–4732, October 2017.
