# Supplementary figures and images for: On the variability of dynamic functional connectivity assessment methods
Source: Gigascience. 2024 Apr 8;13:giae009. doi: 10.1093/gigascience/giae009 (PMC11000510; doi:10.1093/gigascience/giae009)

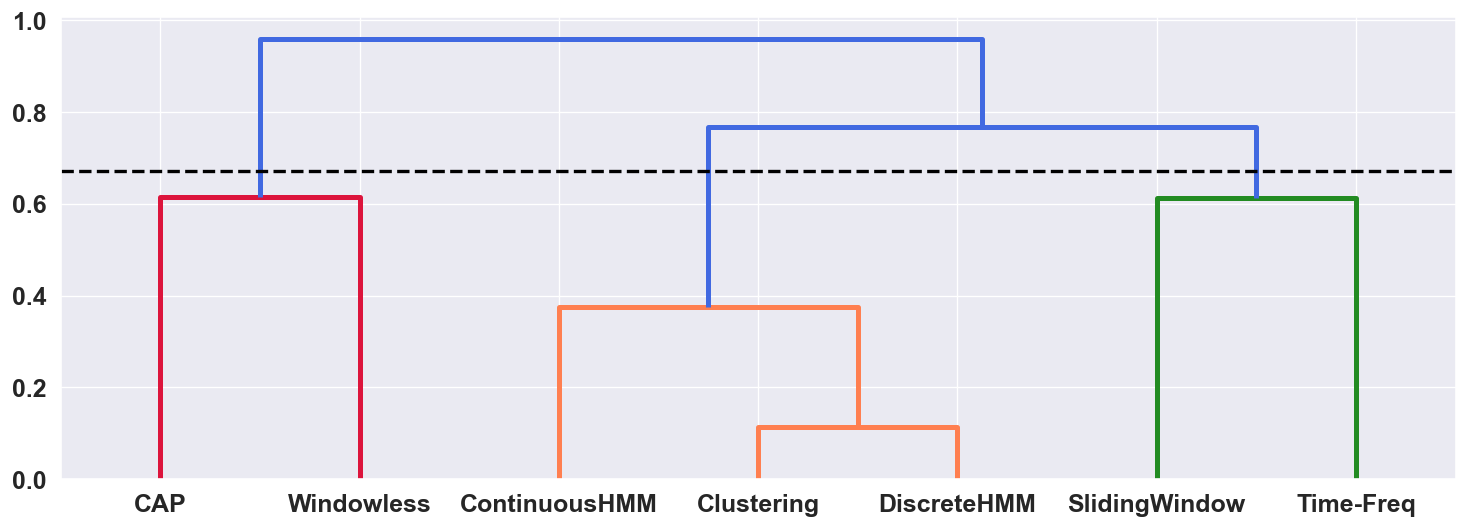

Supplement: giae009_Supplemental_Files [file giae009_supplemental_files.zip › Supplementary Files/SuppFigures/Supplementary_Fig_S21.png]

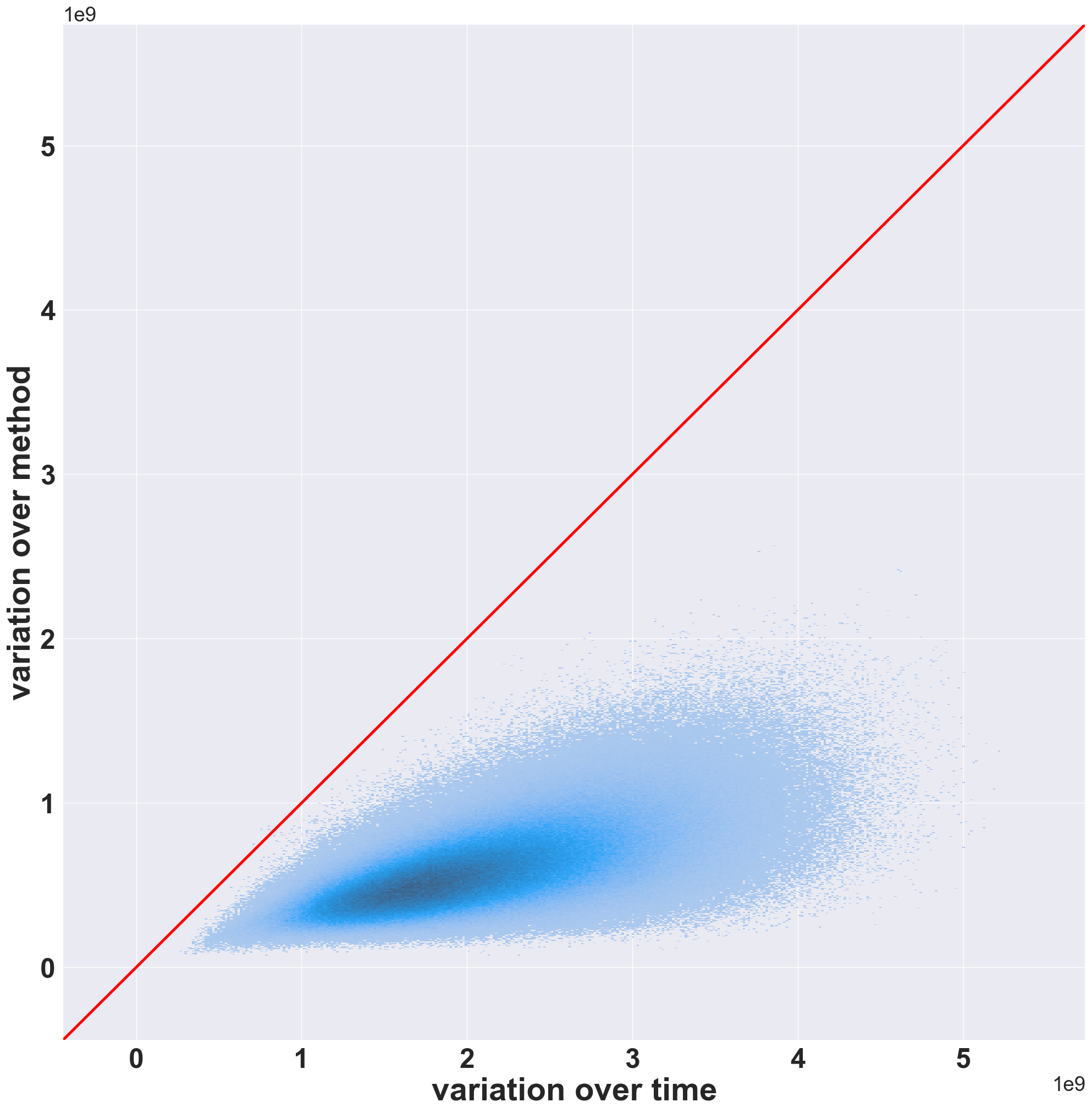

Supplement: giae009_Supplemental_Files [file giae009_supplemental_files.zip › Supplementary Files/SuppFigures/Supplementary_Fig_S35-Group1.png]

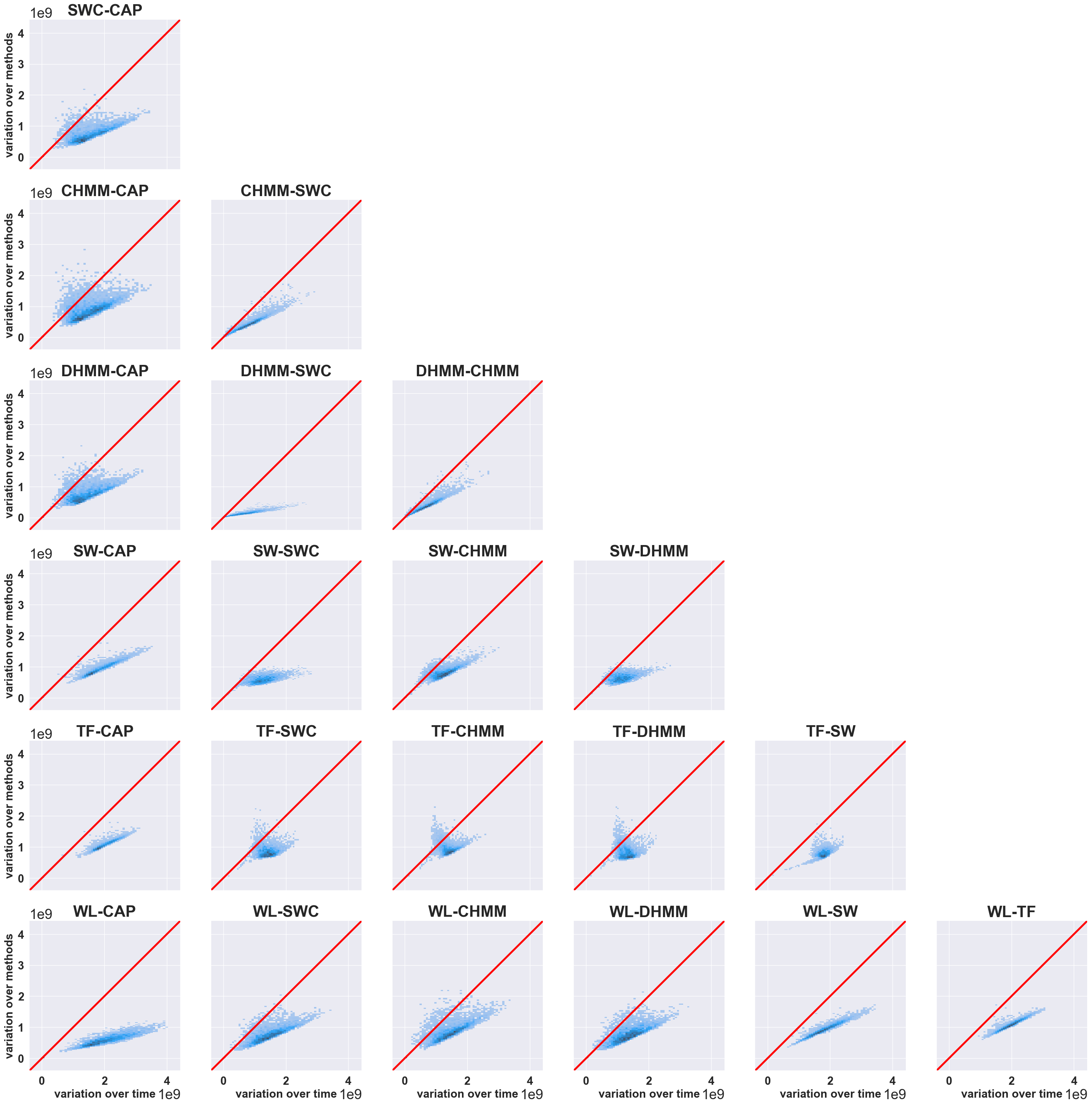

Supplement: giae009_Supplemental_Files [file giae009_supplemental_files.zip › Supplementary Files/SuppFigures/Supplementary_Fig_S34.png]

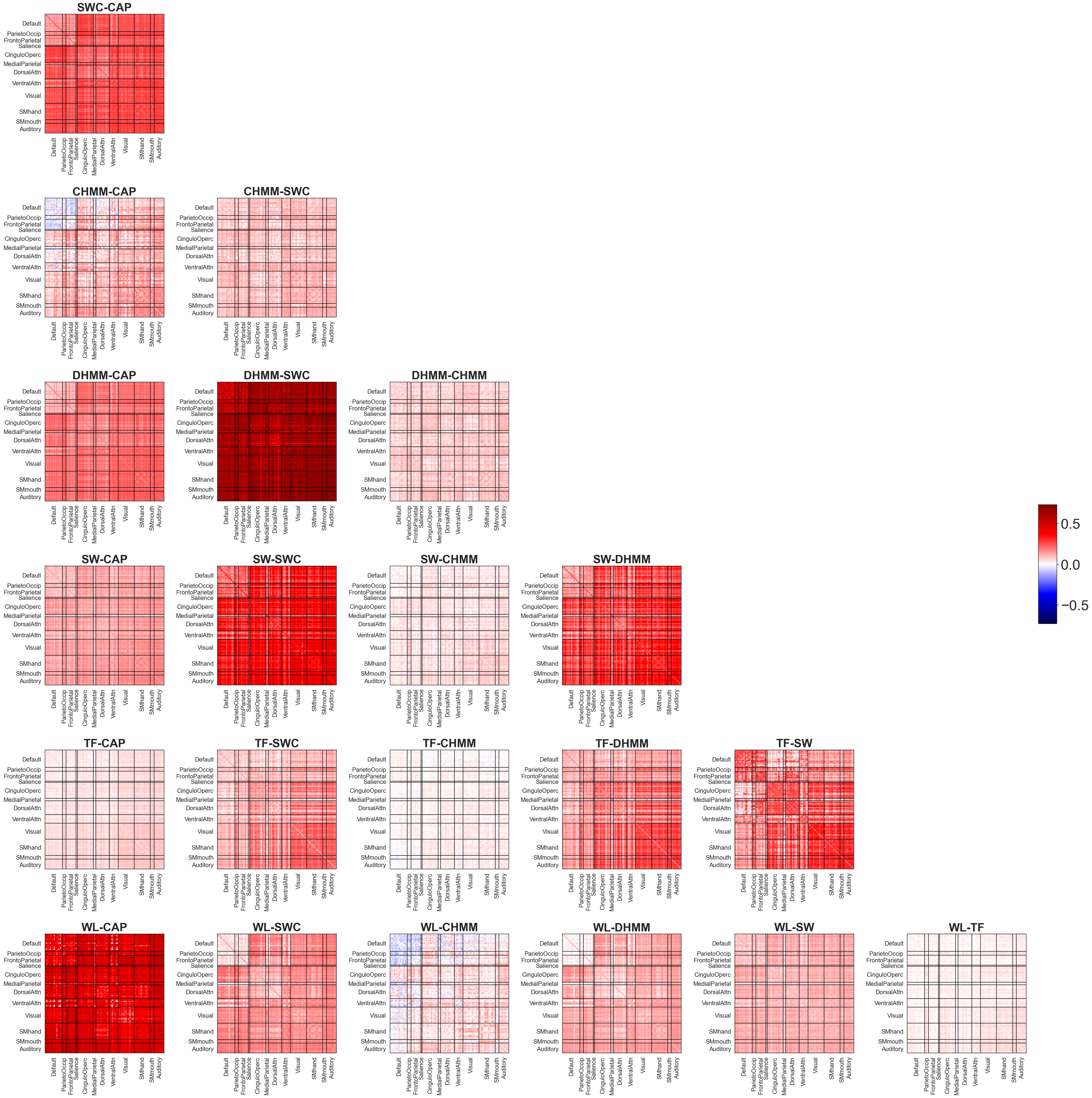

Supplement: giae009_Supplemental_Files [file giae009_supplemental_files.zip › Supplementary Files/SuppFigures/Supplementary_Fig_S20.png]

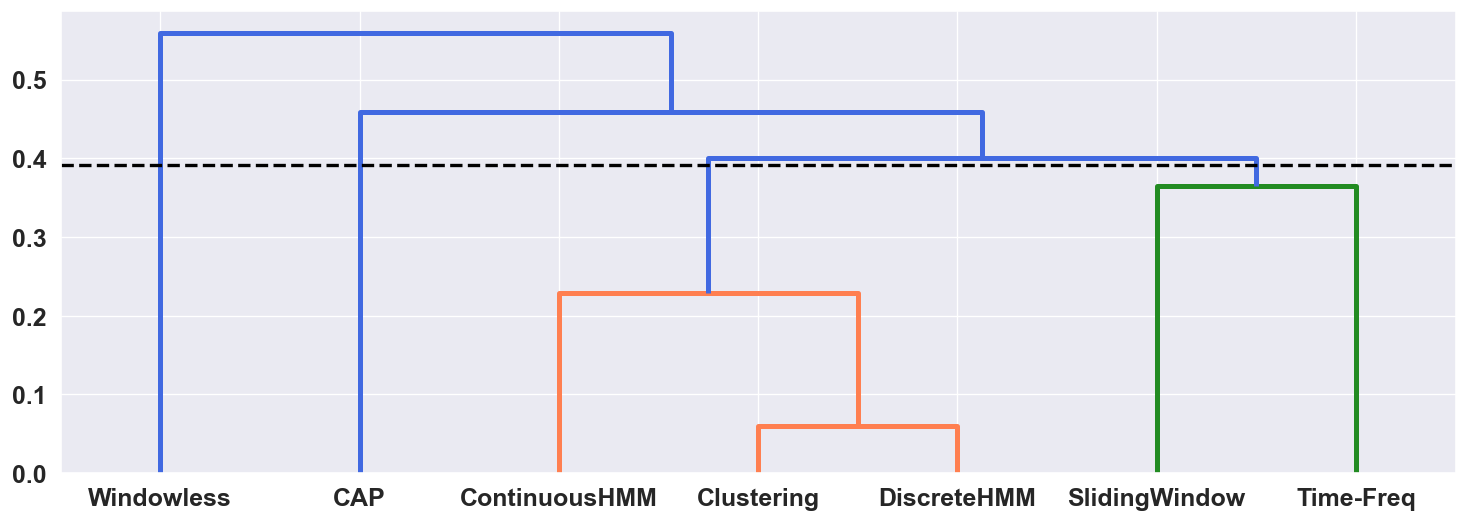

Supplement: giae009_Supplemental_Files [file giae009_supplemental_files.zip › Supplementary Files/SuppFigures/Supplementary_Fig_S22.png]

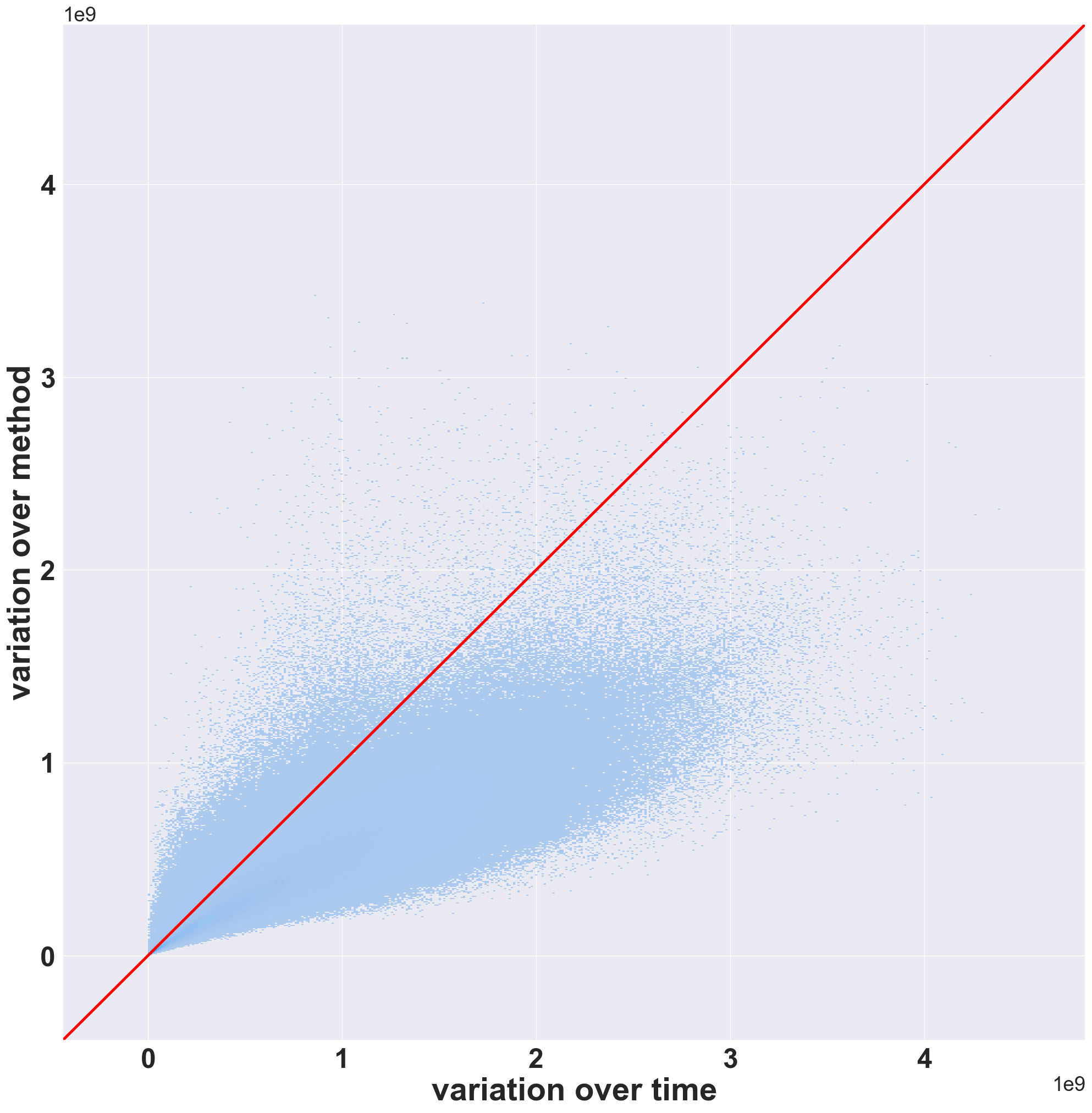

Supplement: giae009_Supplemental_Files [file giae009_supplemental_files.zip › Supplementary Files/SuppFigures/Supplementary_Fig_S35-Group2.png]

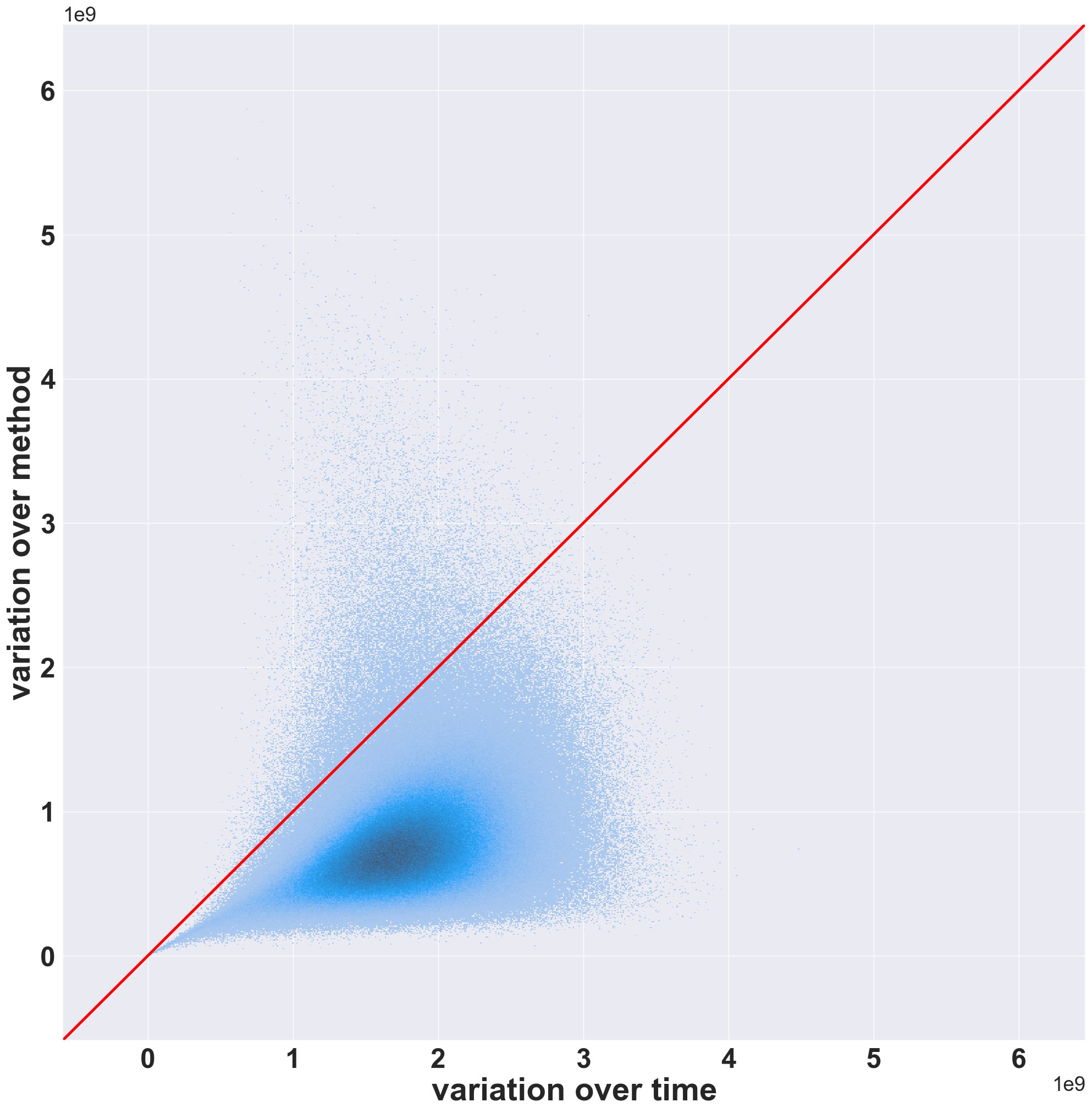

Supplement: giae009_Supplemental_Files [file giae009_supplemental_files.zip › Supplementary Files/SuppFigures/Supplementary_Fig_S35-Group3.png]

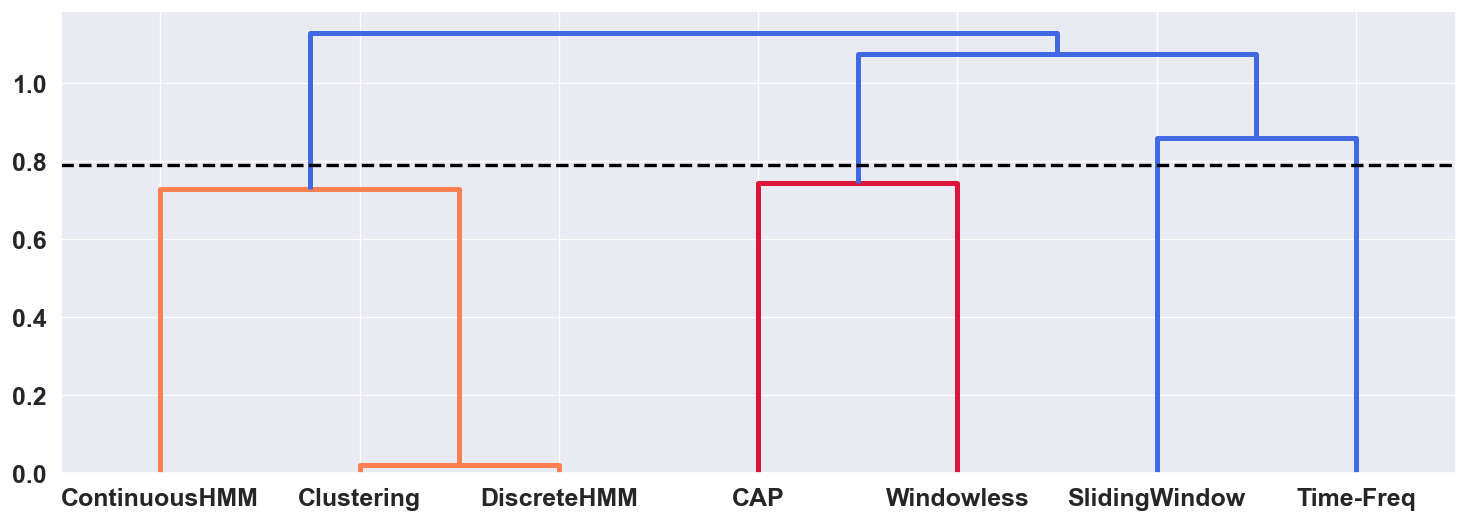

Supplement: giae009_Supplemental_Files [file giae009_supplemental_files.zip › Supplementary Files/SuppFigures/Supplementary_Fig_S23.png]

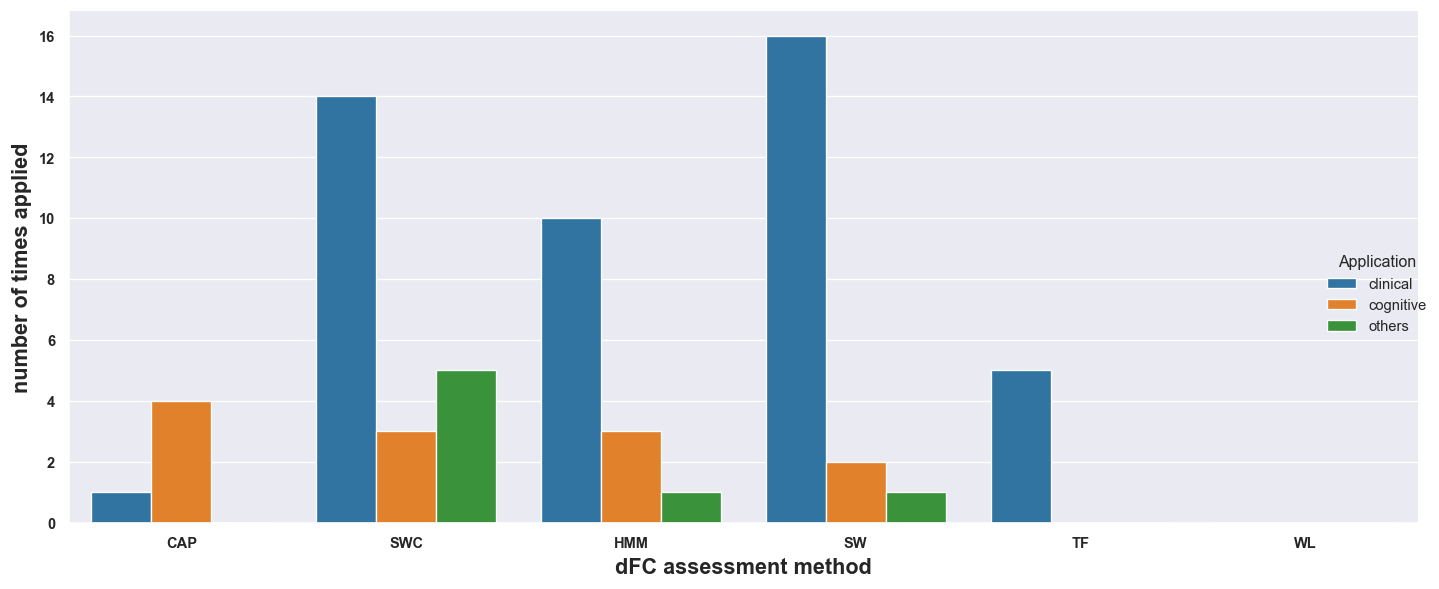

Supplement: giae009_Supplemental_Files [file giae009_supplemental_files.zip › Supplementary Files/SuppFigures/Supplementary_Fig_S37.png]

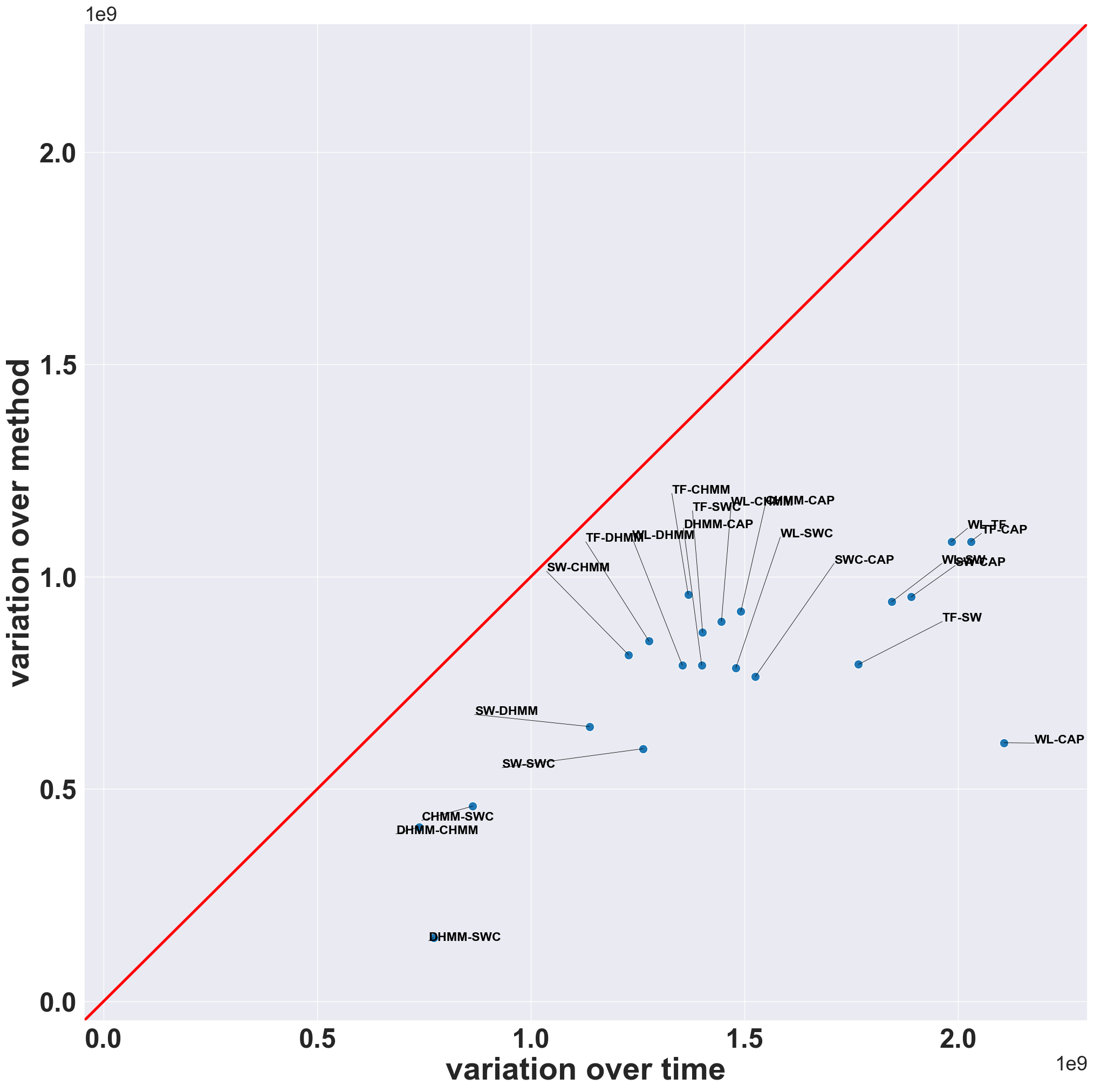

Supplement: giae009_Supplemental_Files [file giae009_supplemental_files.zip › Supplementary Files/SuppFigures/Supplementary_Fig_S33.png]

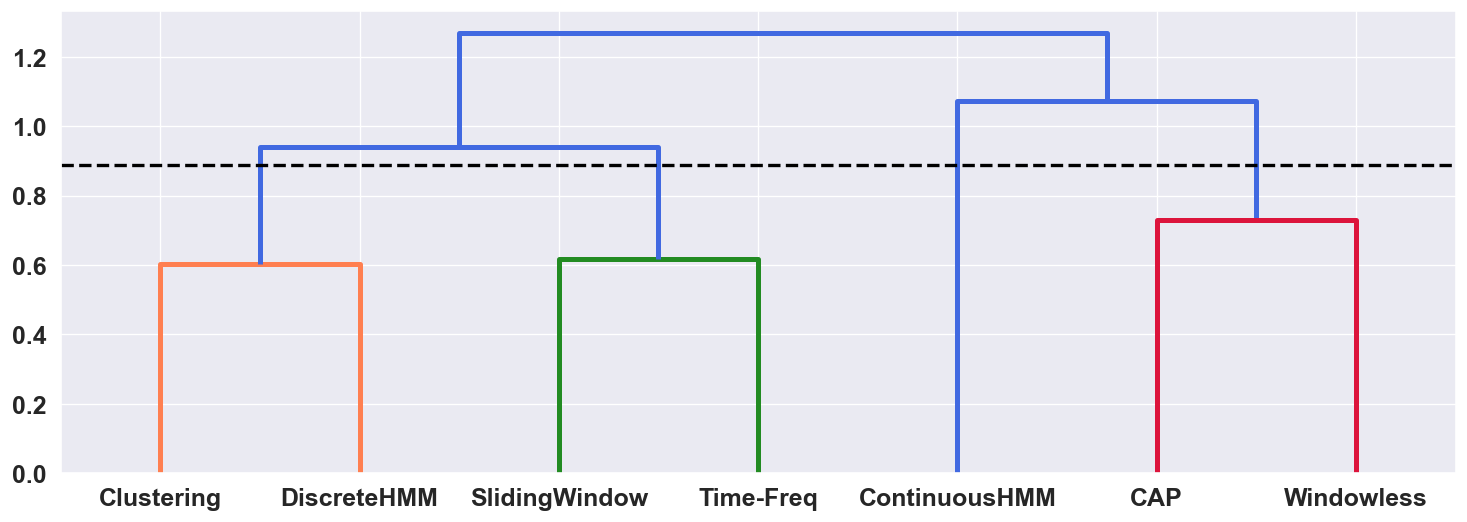

Supplement: giae009_Supplemental_Files [file giae009_supplemental_files.zip › Supplementary Files/SuppFigures/Supplementary_Fig_S27.png]

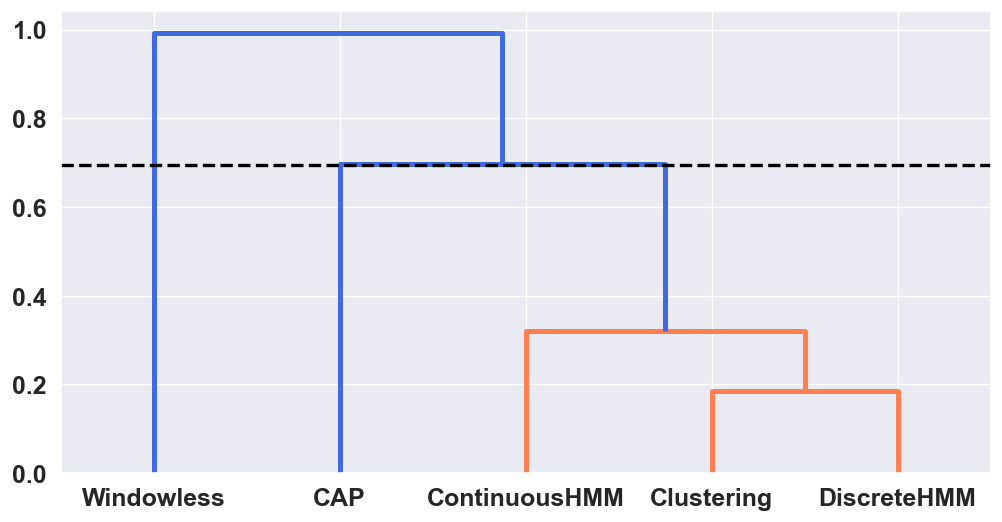

Supplement: giae009_Supplemental_Files [file giae009_supplemental_files.zip › Supplementary Files/SuppFigures/Supplementary_Fig_S26.png]

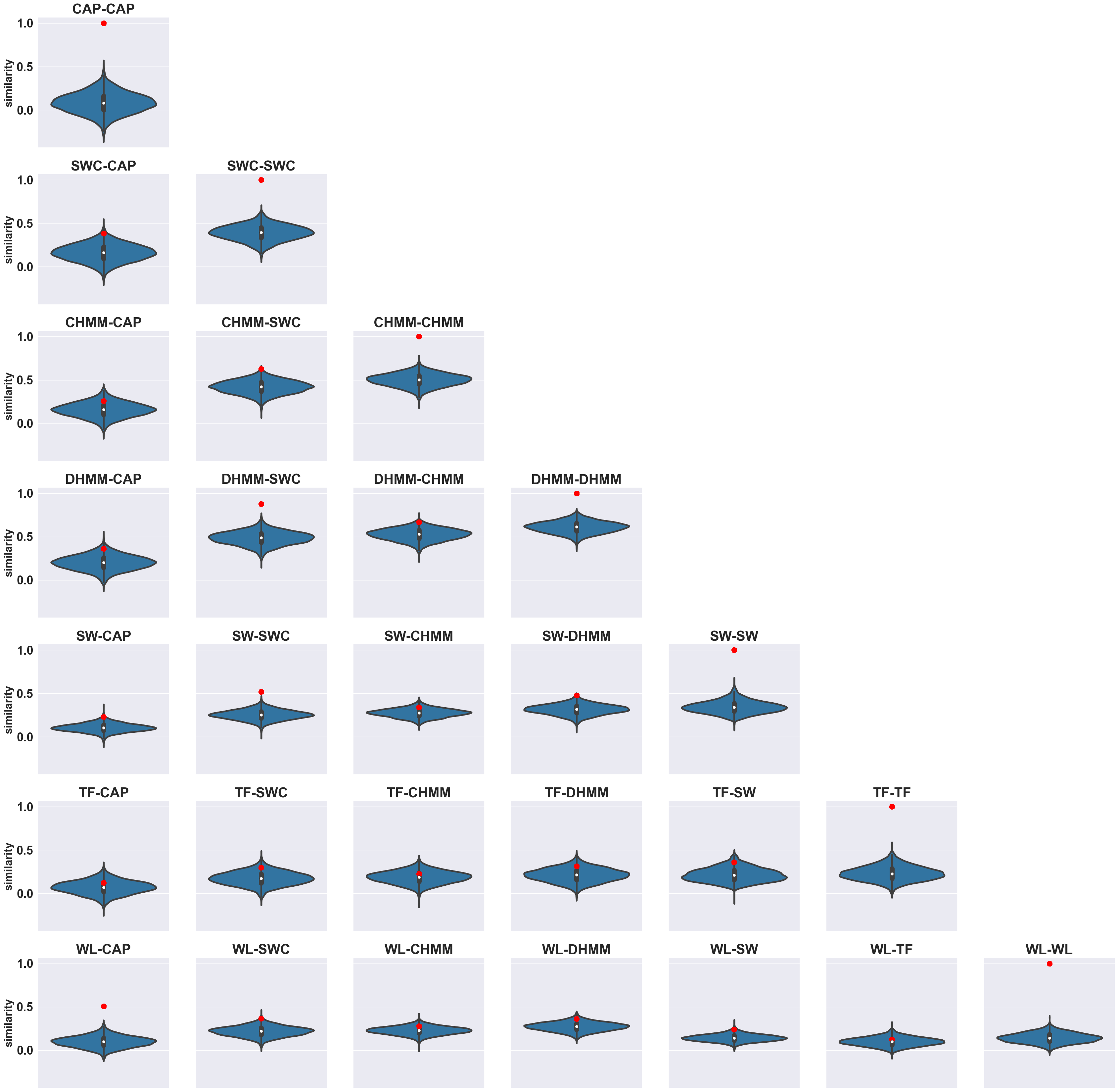

Supplement: giae009_Supplemental_Files [file giae009_supplemental_files.zip › Supplementary Files/SuppFigures/Supplementary_Fig_S32.png]

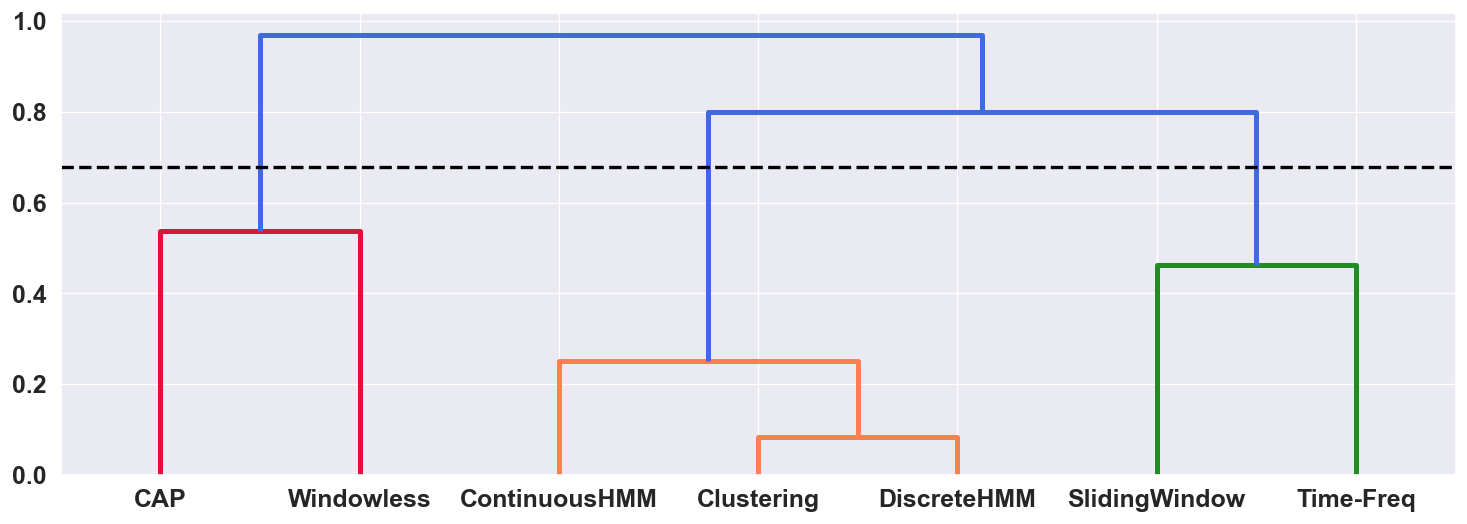

Supplement: giae009_Supplemental_Files [file giae009_supplemental_files.zip › Supplementary Files/SuppFigures/Supplementary_Fig_S24.png]

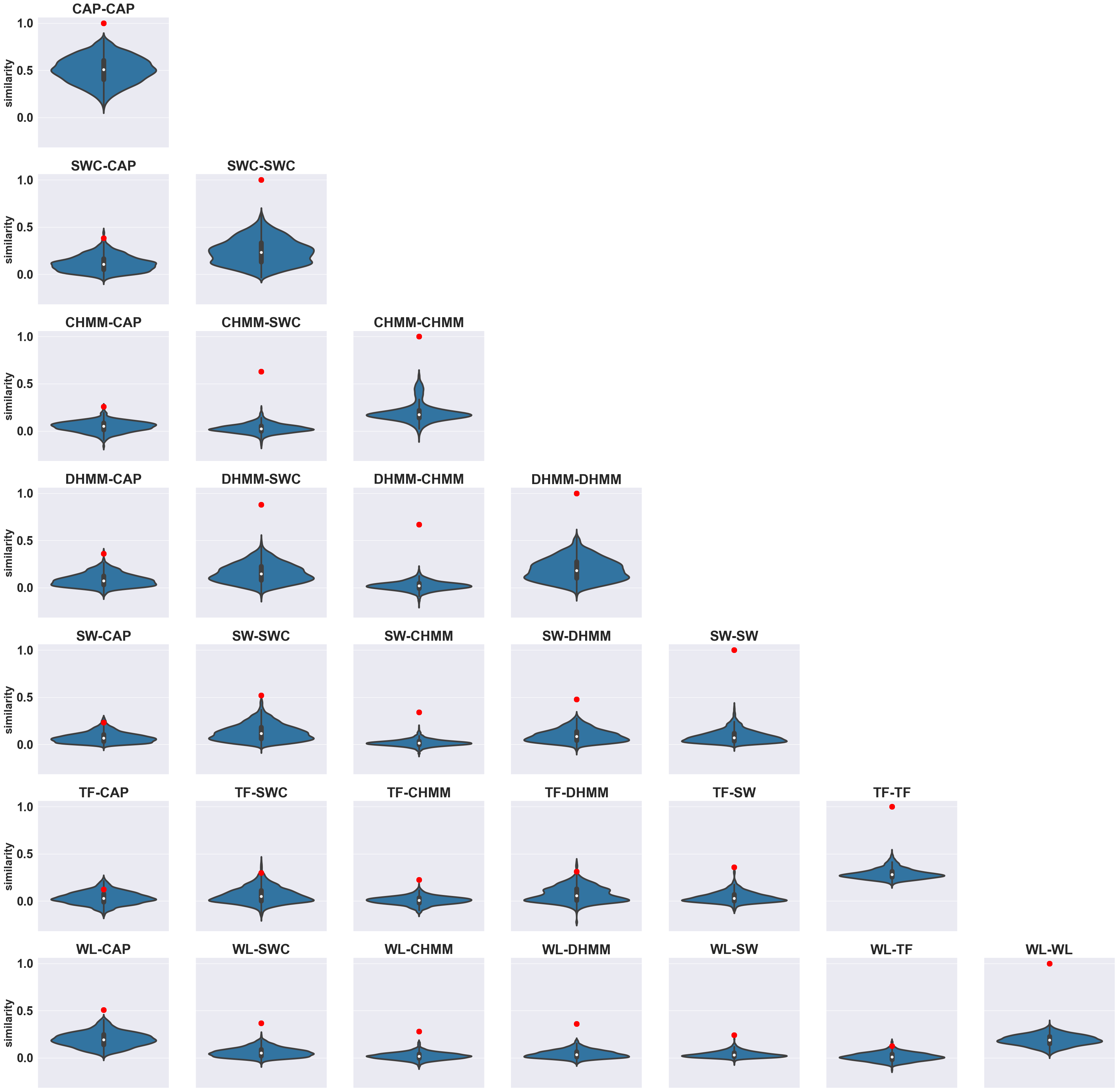

Supplement: giae009_Supplemental_Files [file giae009_supplemental_files.zip › Supplementary Files/SuppFigures/Supplementary_Fig_S30.png]

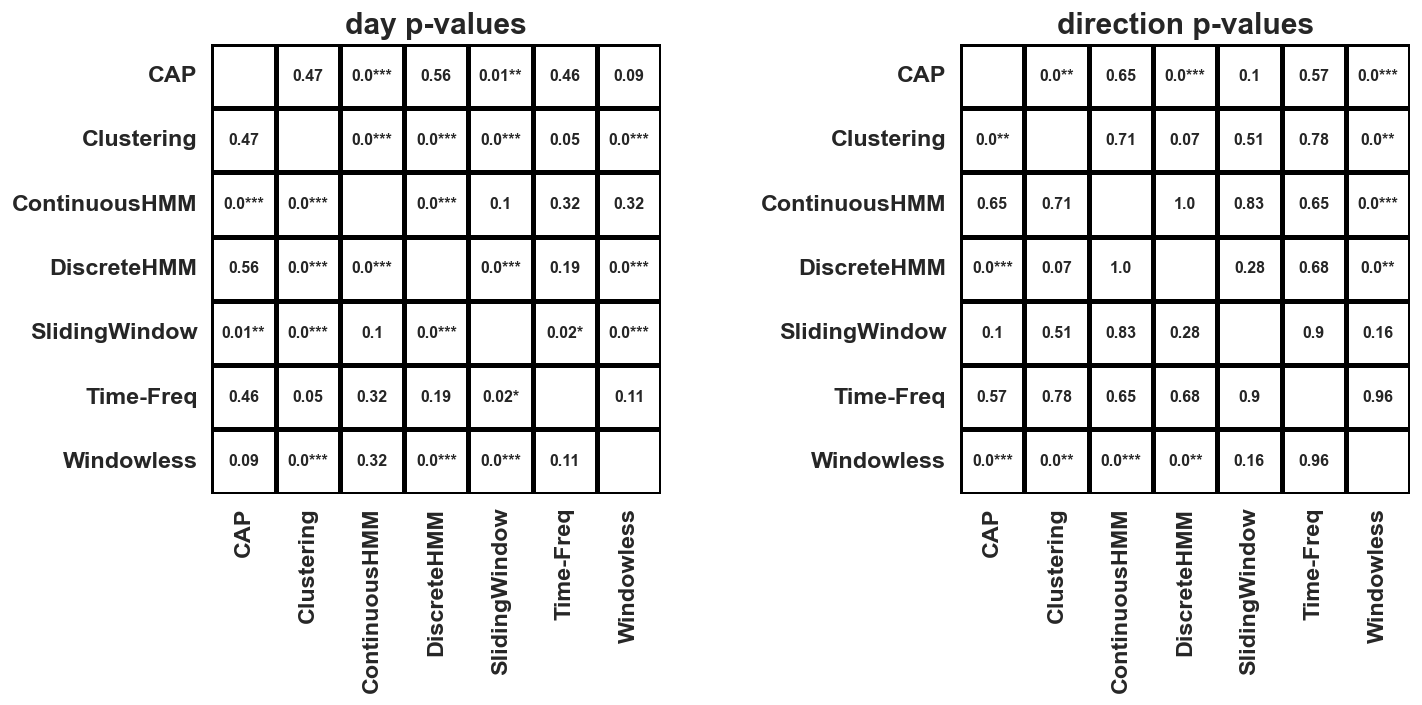

Supplement: giae009_Supplemental_Files [file giae009_supplemental_files.zip › Supplementary Files/SuppFigures/Supplementary_Fig_S19.png]

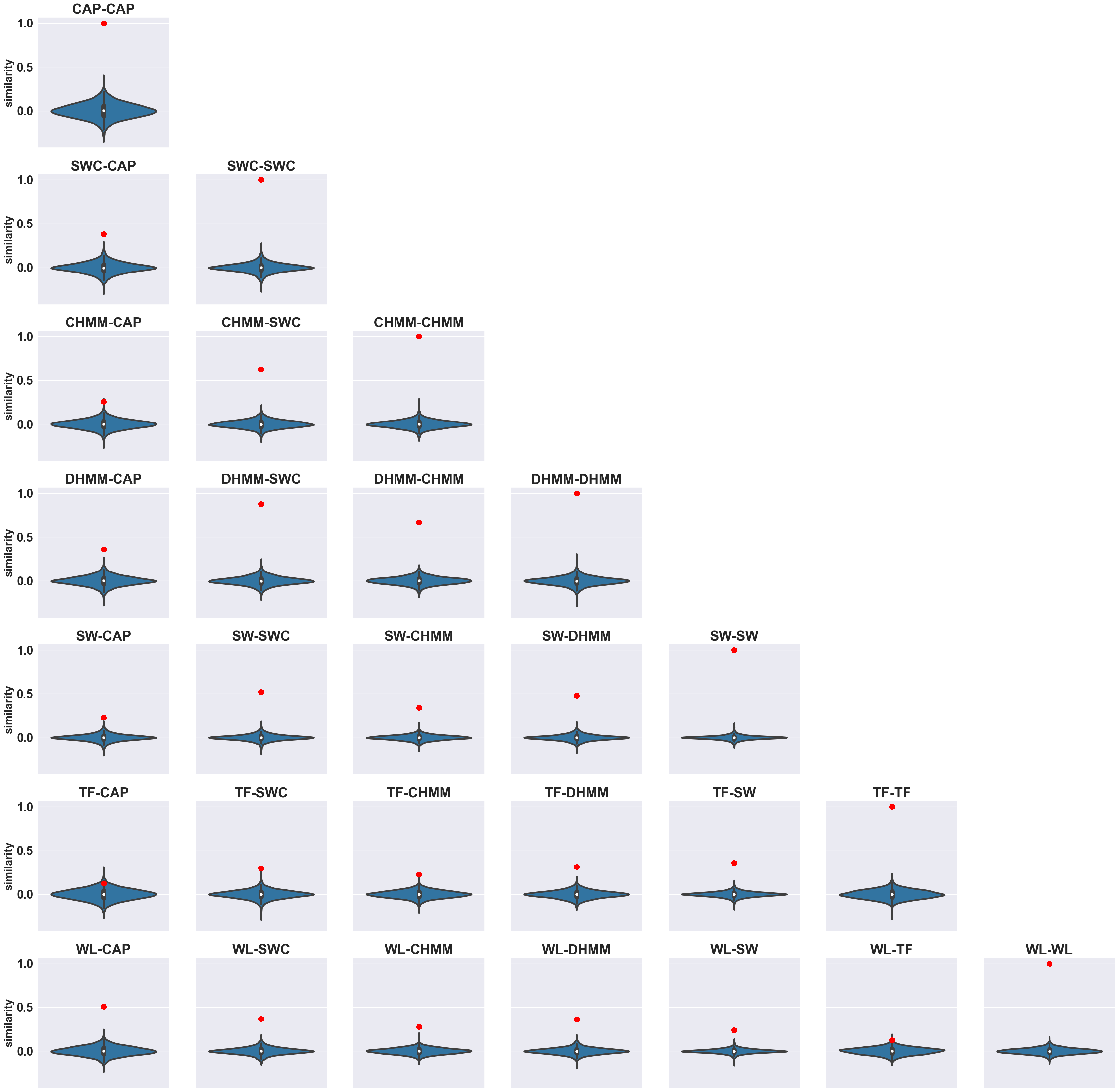

Supplement: giae009_Supplemental_Files [file giae009_supplemental_files.zip › Supplementary Files/SuppFigures/Supplementary_Fig_S31.png]

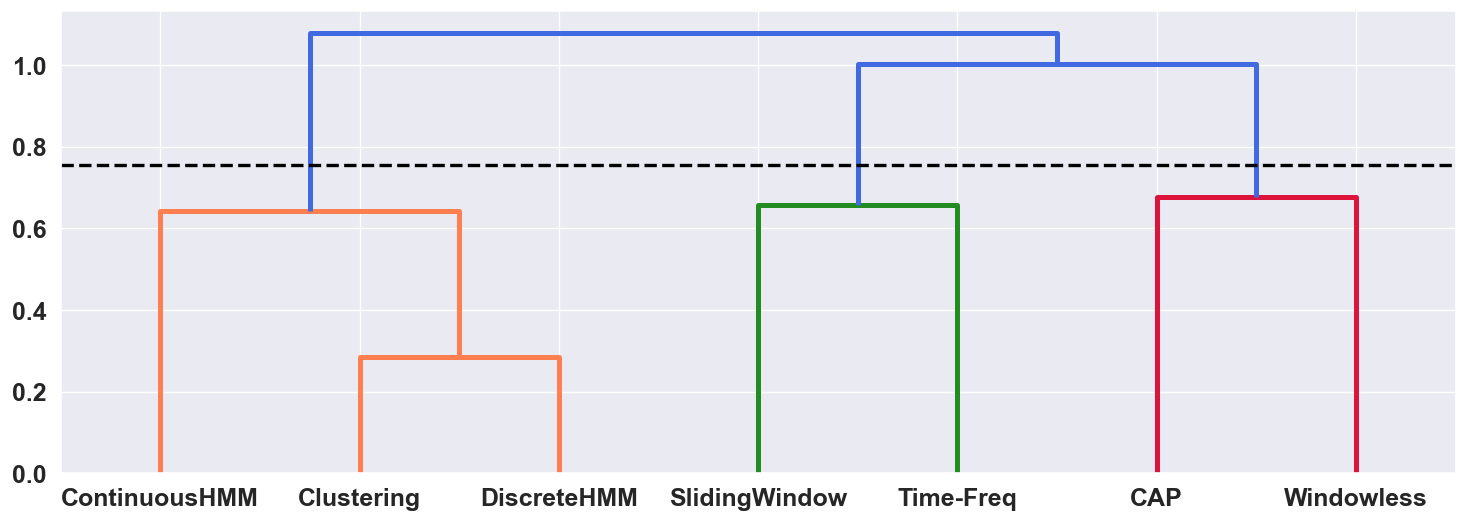

Supplement: giae009_Supplemental_Files [file giae009_supplemental_files.zip › Supplementary Files/SuppFigures/Supplementary_Fig_S25.png]

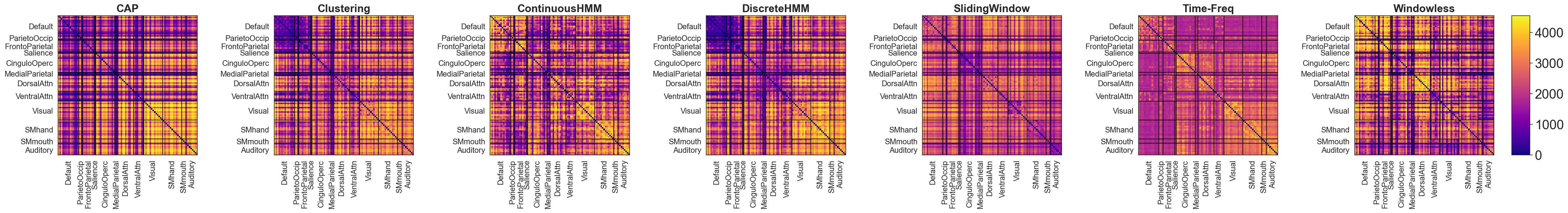

Supplement: giae009_Supplemental_Files [file giae009_supplemental_files.zip › Supplementary Files/SuppFigures/Supplementary_Fig_S8.png]

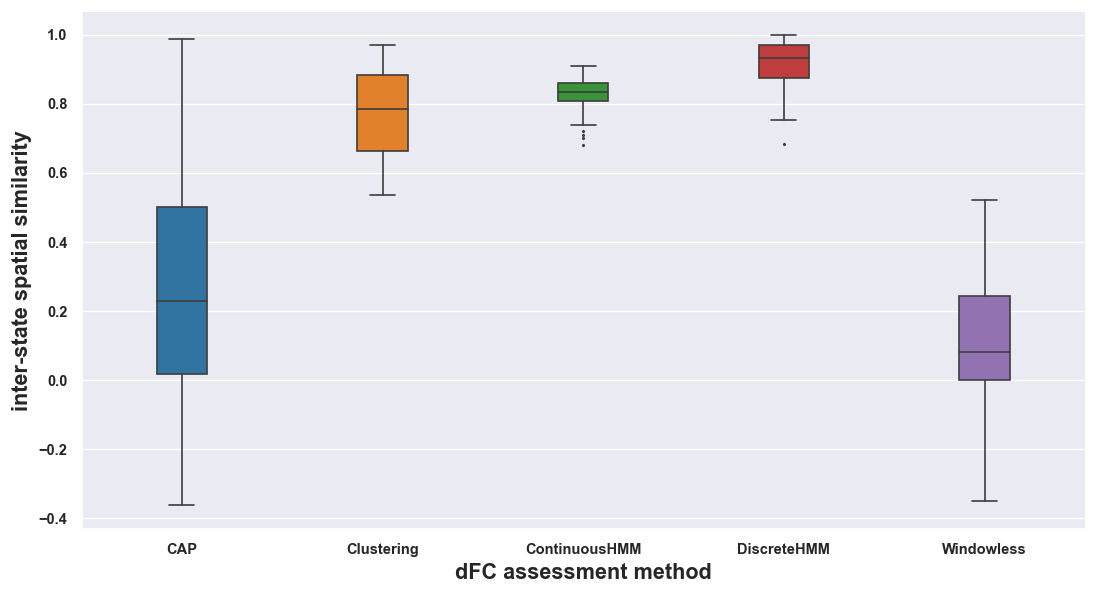

Supplement: giae009_Supplemental_Files [file giae009_supplemental_files.zip › Supplementary Files/SuppFigures/Supplementary_Fig_S9.png]

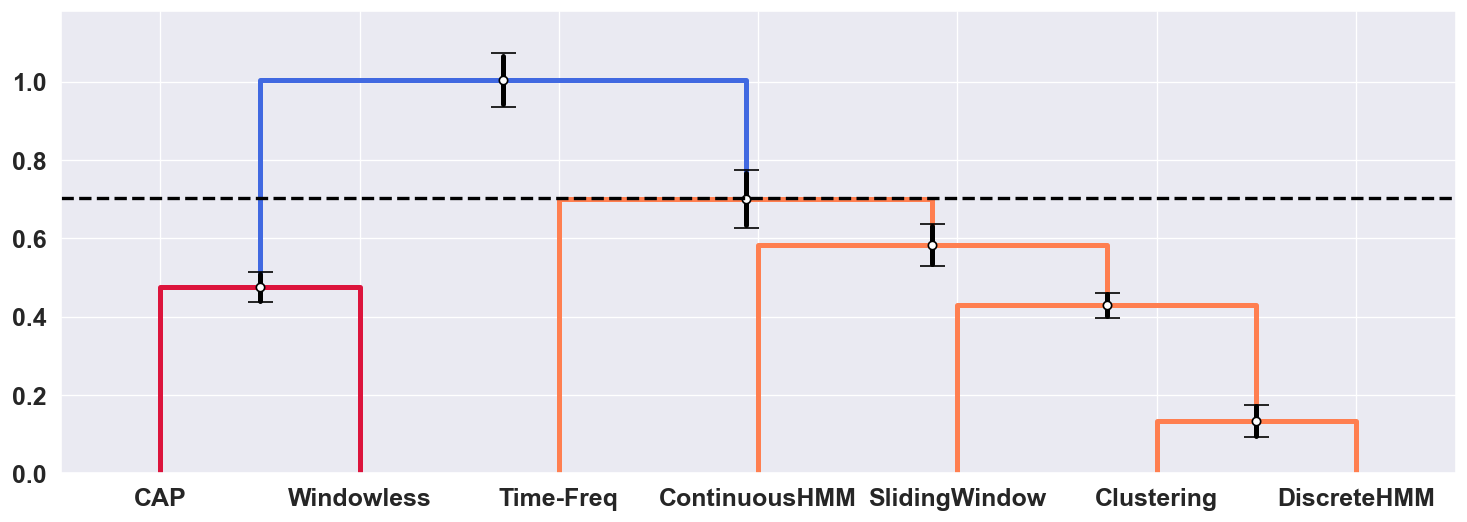

Supplement: giae009_Supplemental_Files [file giae009_supplemental_files.zip › Supplementary Files/SuppFigures/Supplementary_Fig_S18-5.png]

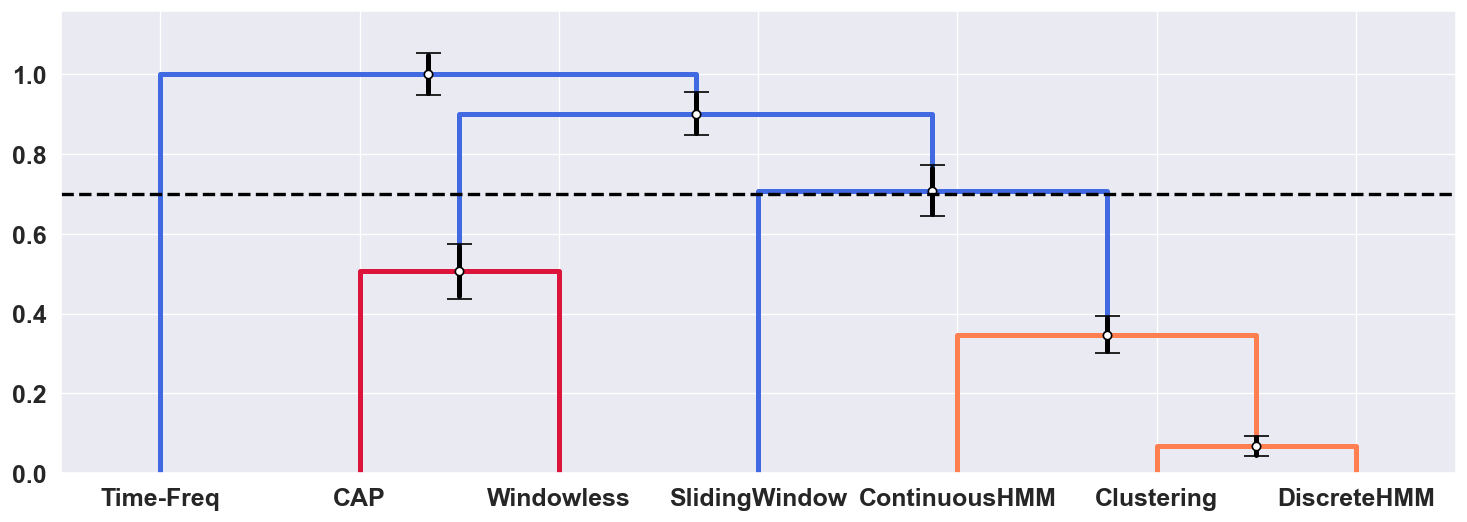

Supplement: giae009_Supplemental_Files [file giae009_supplemental_files.zip › Supplementary Files/SuppFigures/Supplementary_Fig_S18-4.png]

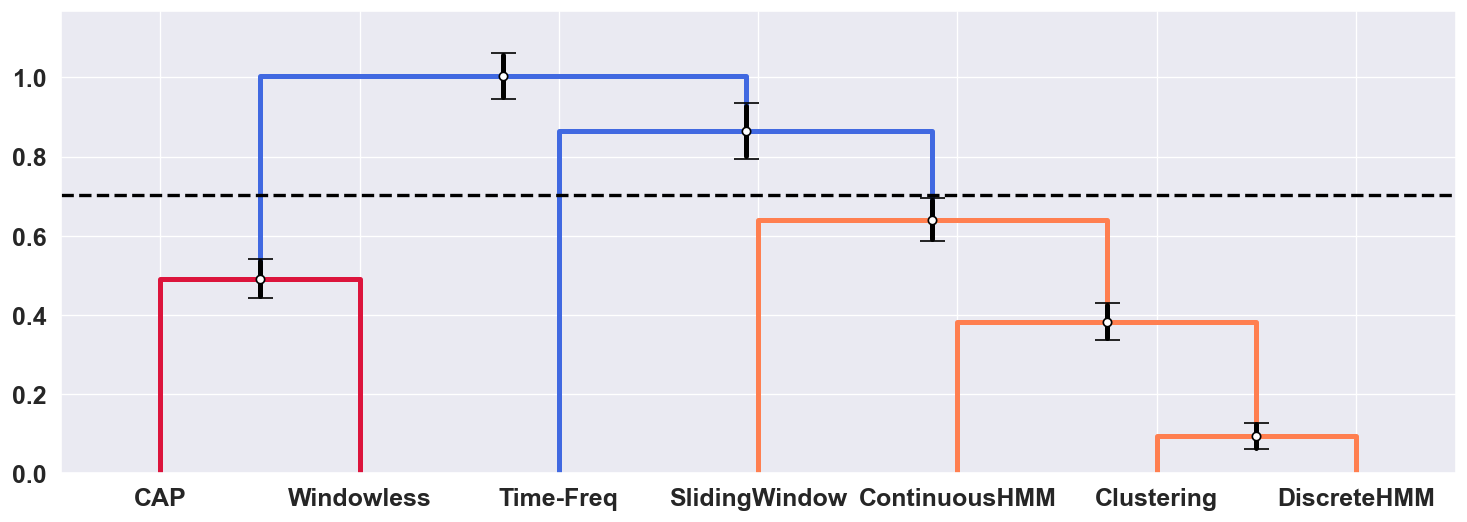

Supplement: giae009_Supplemental_Files [file giae009_supplemental_files.zip › Supplementary Files/SuppFigures/Supplementary_Fig_S18-1.png]

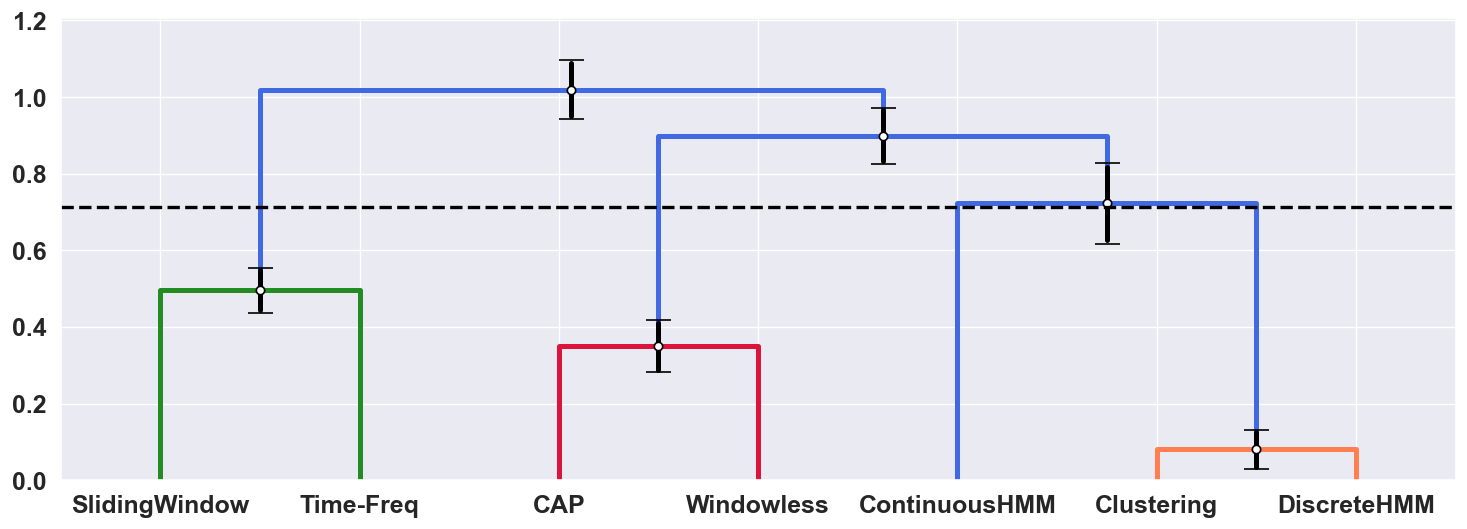

Supplement: giae009_Supplemental_Files [file giae009_supplemental_files.zip › Supplementary Files/SuppFigures/Supplementary_Fig_S18-3.png]

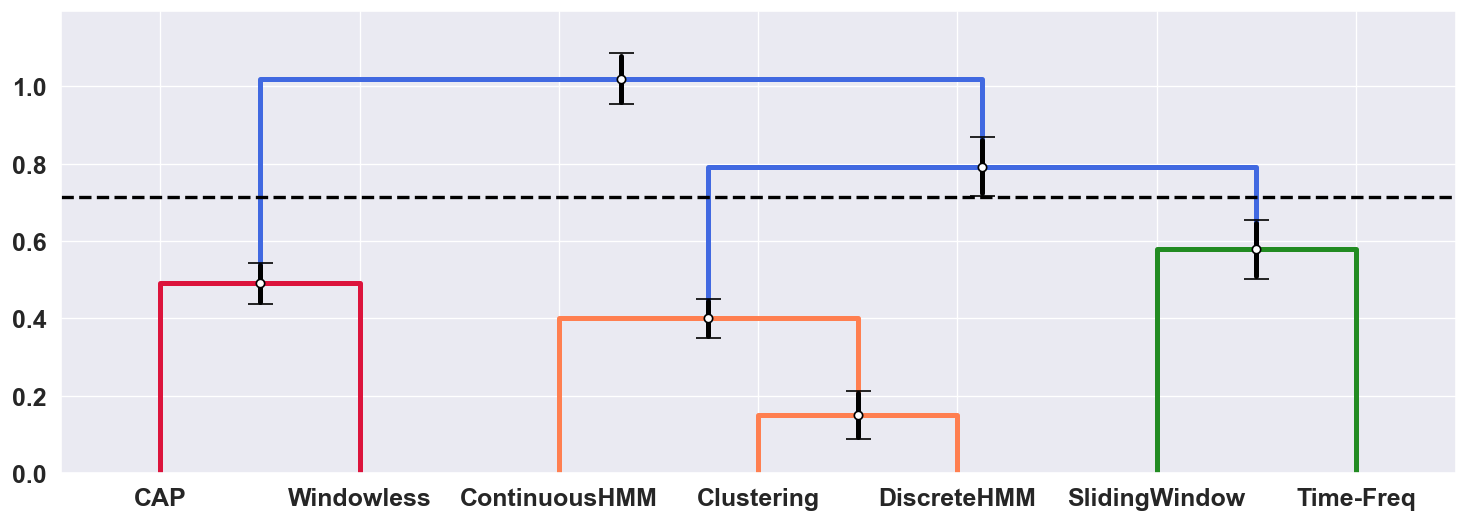

Supplement: giae009_Supplemental_Files [file giae009_supplemental_files.zip › Supplementary Files/SuppFigures/Supplementary_Fig_S18-2.png]

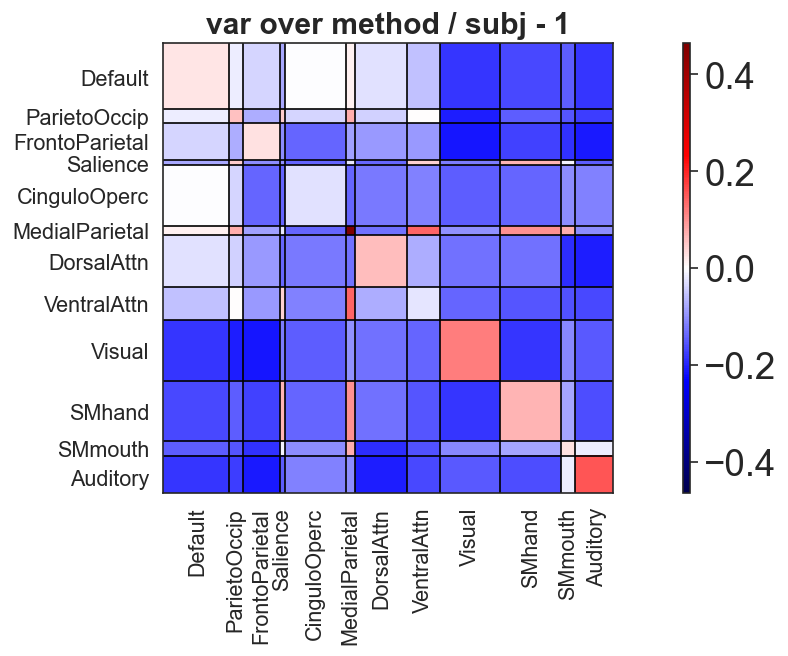

Supplement: giae009_Supplemental_Files [file giae009_supplemental_files.zip › Supplementary Files/SuppFigures/Supplementary_Fig_S36-Right.png]

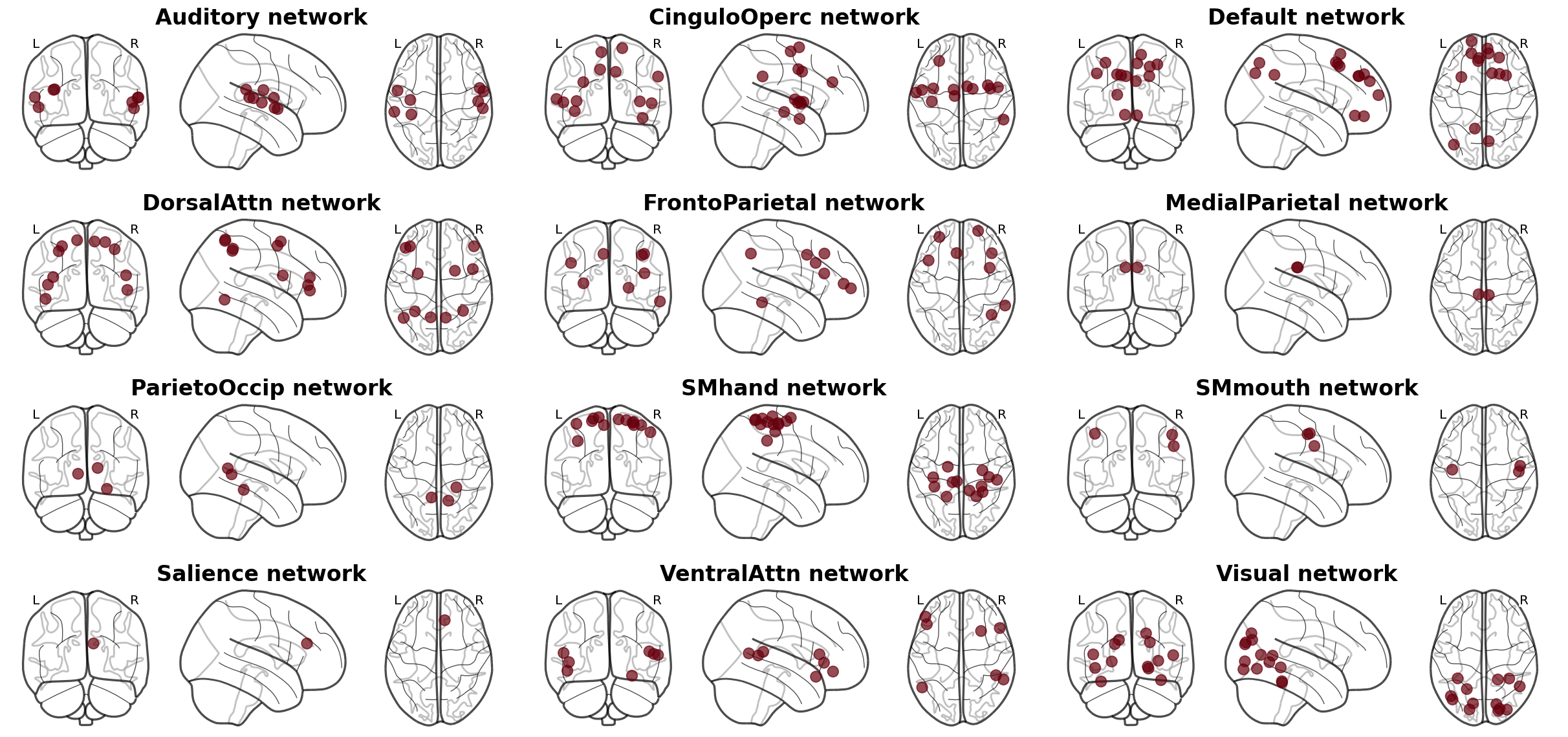

Supplement: giae009_Supplemental_Files [file giae009_supplemental_files.zip › Supplementary Files/SuppFigures/Supplementary_Fig_S1.png]

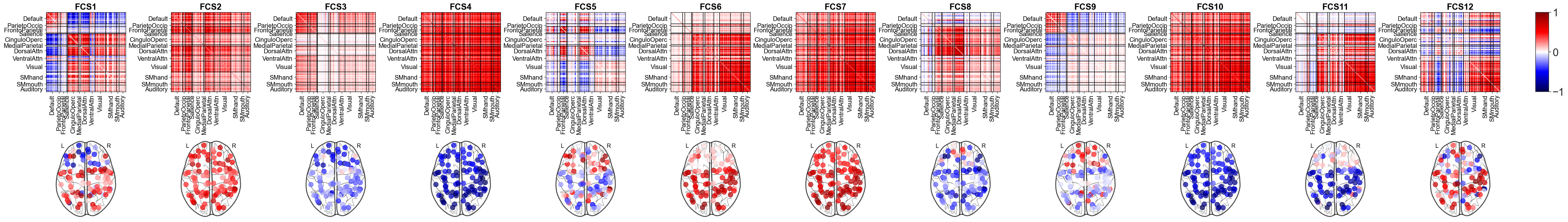

Supplement: giae009_Supplemental_Files [file giae009_supplemental_files.zip › Supplementary Files/SuppFigures/Supplementary_Fig_S2.png]

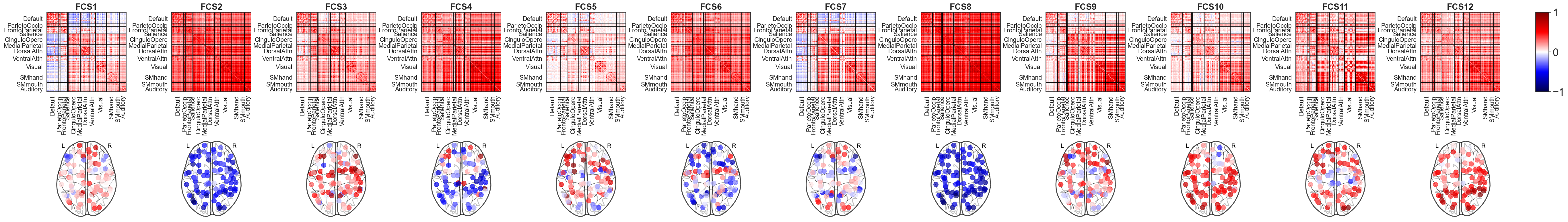

Supplement: giae009_Supplemental_Files [file giae009_supplemental_files.zip › Supplementary Files/SuppFigures/Supplementary_Fig_S3.png]

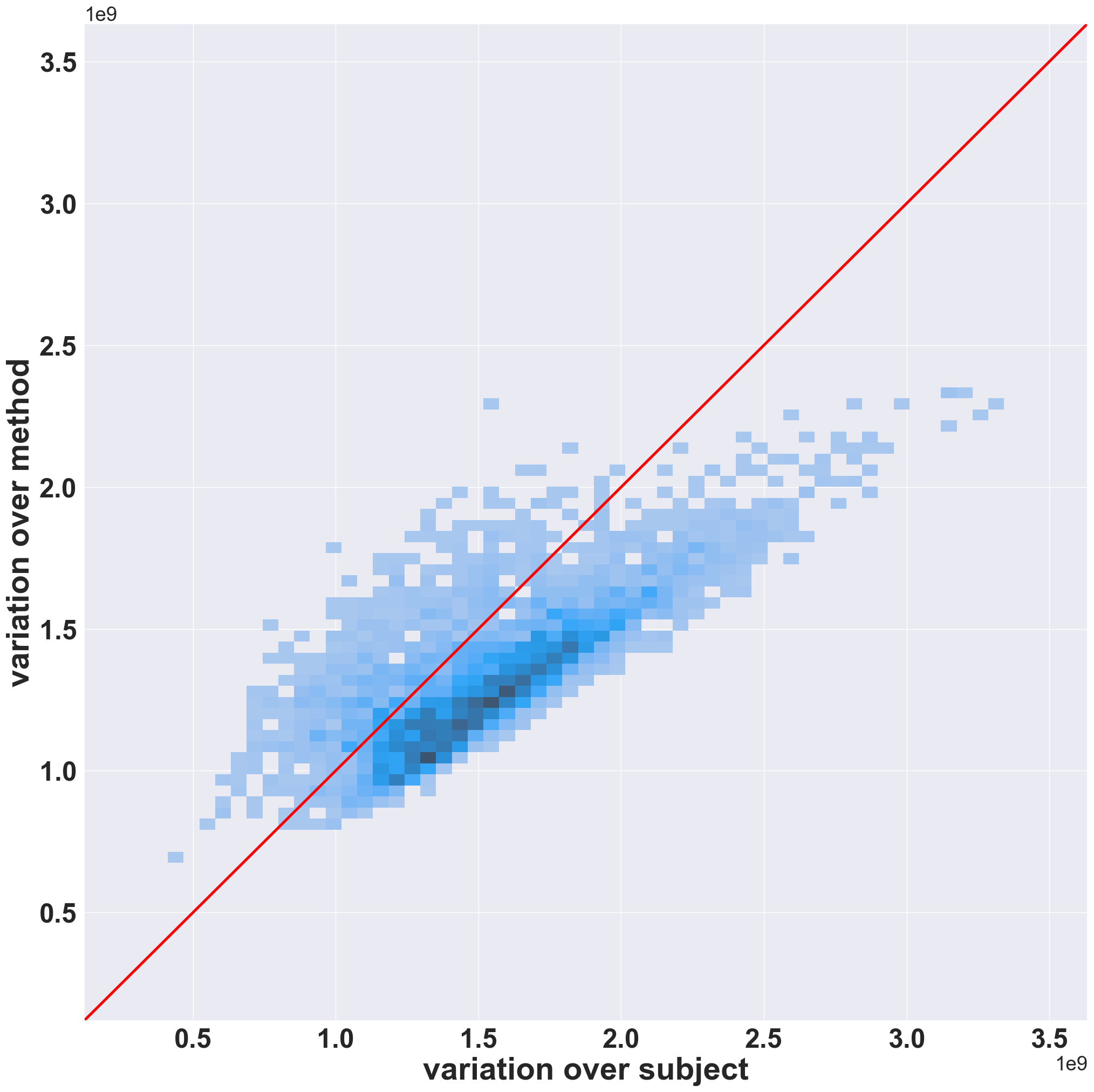

Supplement: giae009_Supplemental_Files [file giae009_supplemental_files.zip › Supplementary Files/SuppFigures/Supplementary_Fig_S36-Left.png]

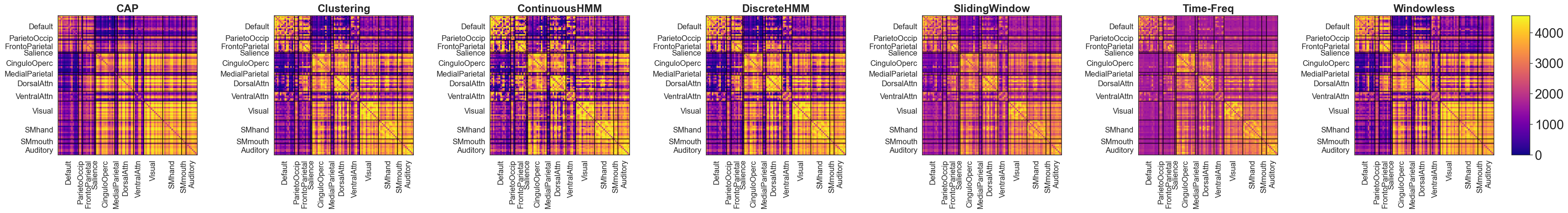

Supplement: giae009_Supplemental_Files [file giae009_supplemental_files.zip › Supplementary Files/SuppFigures/Supplementary_Fig_S7.png]

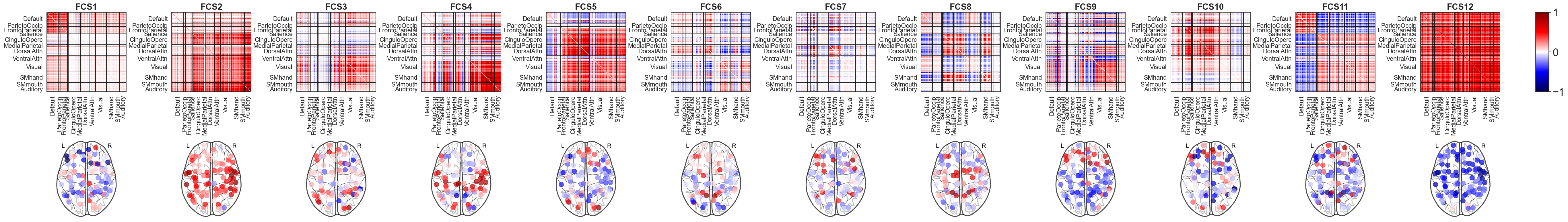

Supplement: giae009_Supplemental_Files [file giae009_supplemental_files.zip › Supplementary Files/SuppFigures/Supplementary_Fig_S6.png]

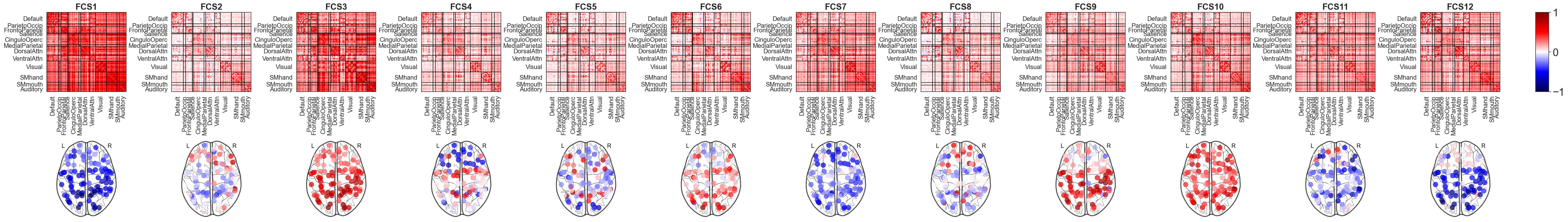

Supplement: giae009_Supplemental_Files [file giae009_supplemental_files.zip › Supplementary Files/SuppFigures/Supplementary_Fig_S4.png]

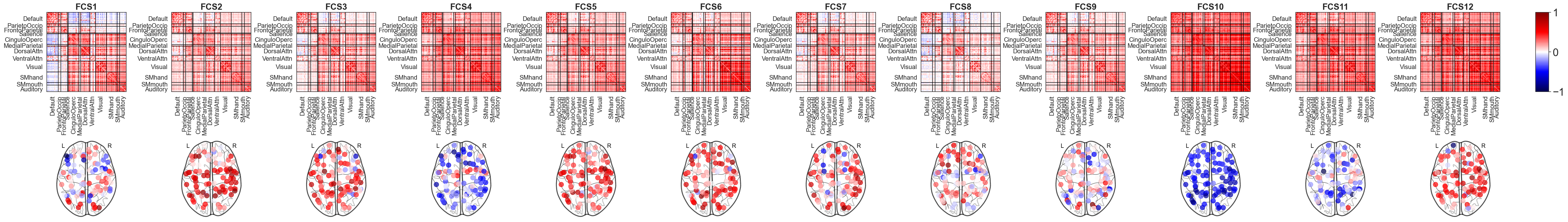

Supplement: giae009_Supplemental_Files [file giae009_supplemental_files.zip › Supplementary Files/SuppFigures/Supplementary_Fig_S5.png]

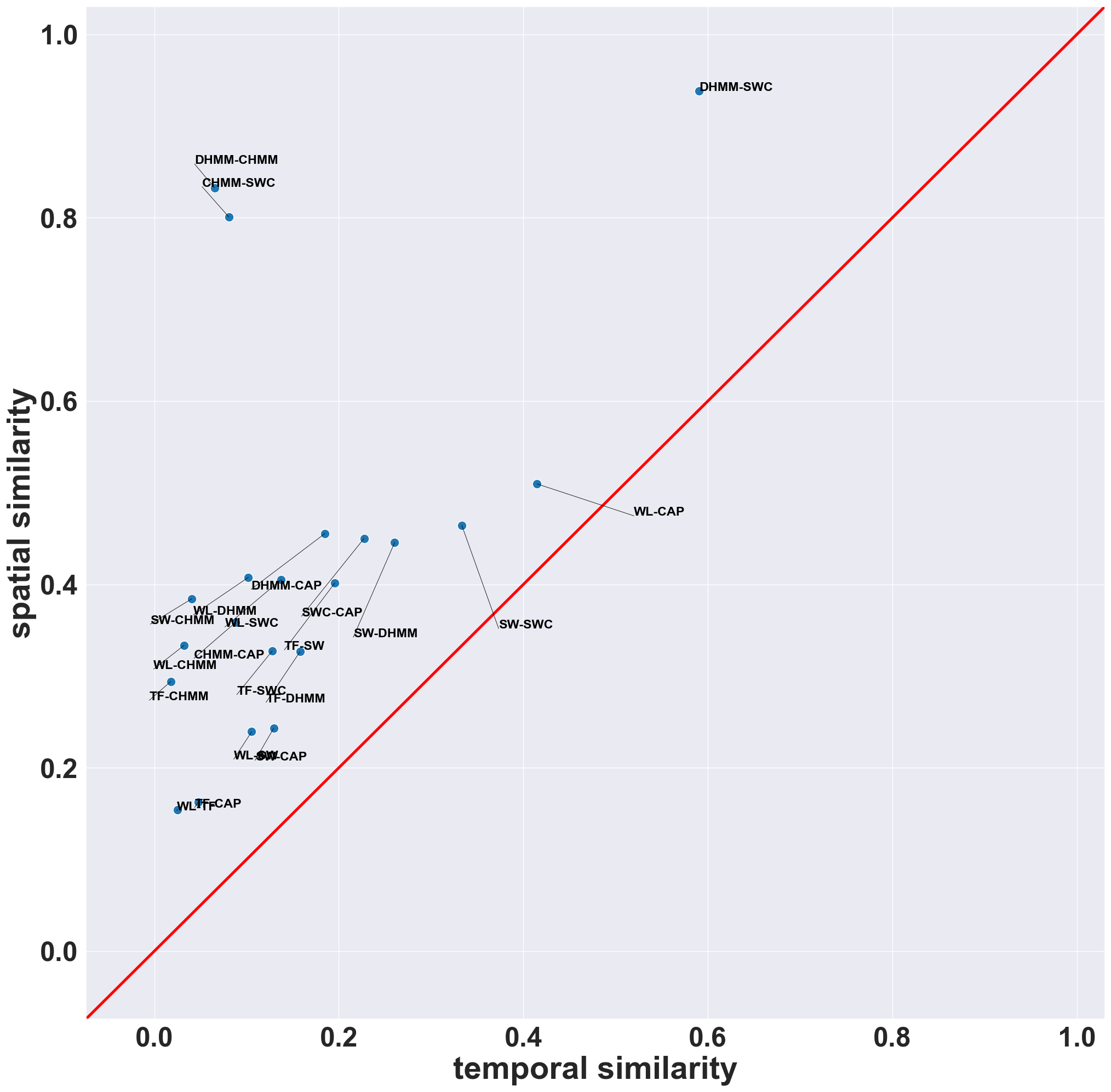

Supplement: giae009_Supplemental_Files [file giae009_supplemental_files.zip › Supplementary Files/SuppFigures/Supplementary_Fig_S28.png]

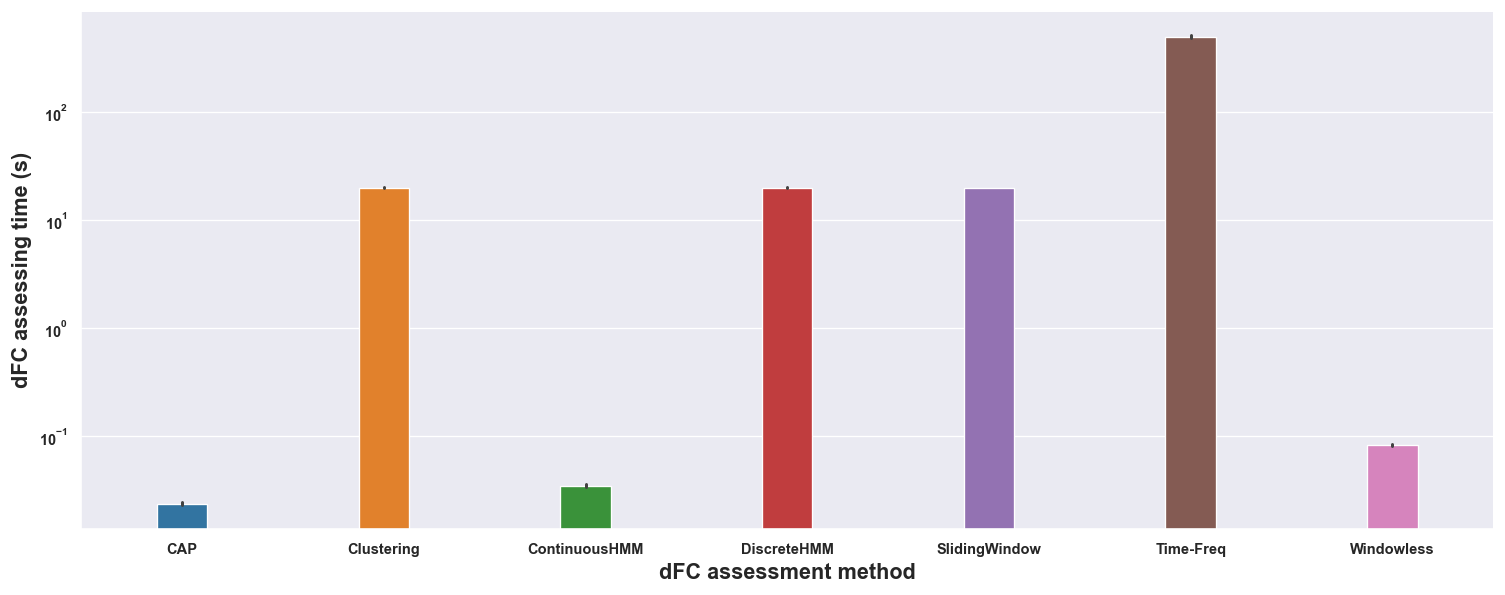

Supplement: giae009_Supplemental_Files [file giae009_supplemental_files.zip › Supplementary Files/SuppFigures/Supplementary_Fig_S14.png]

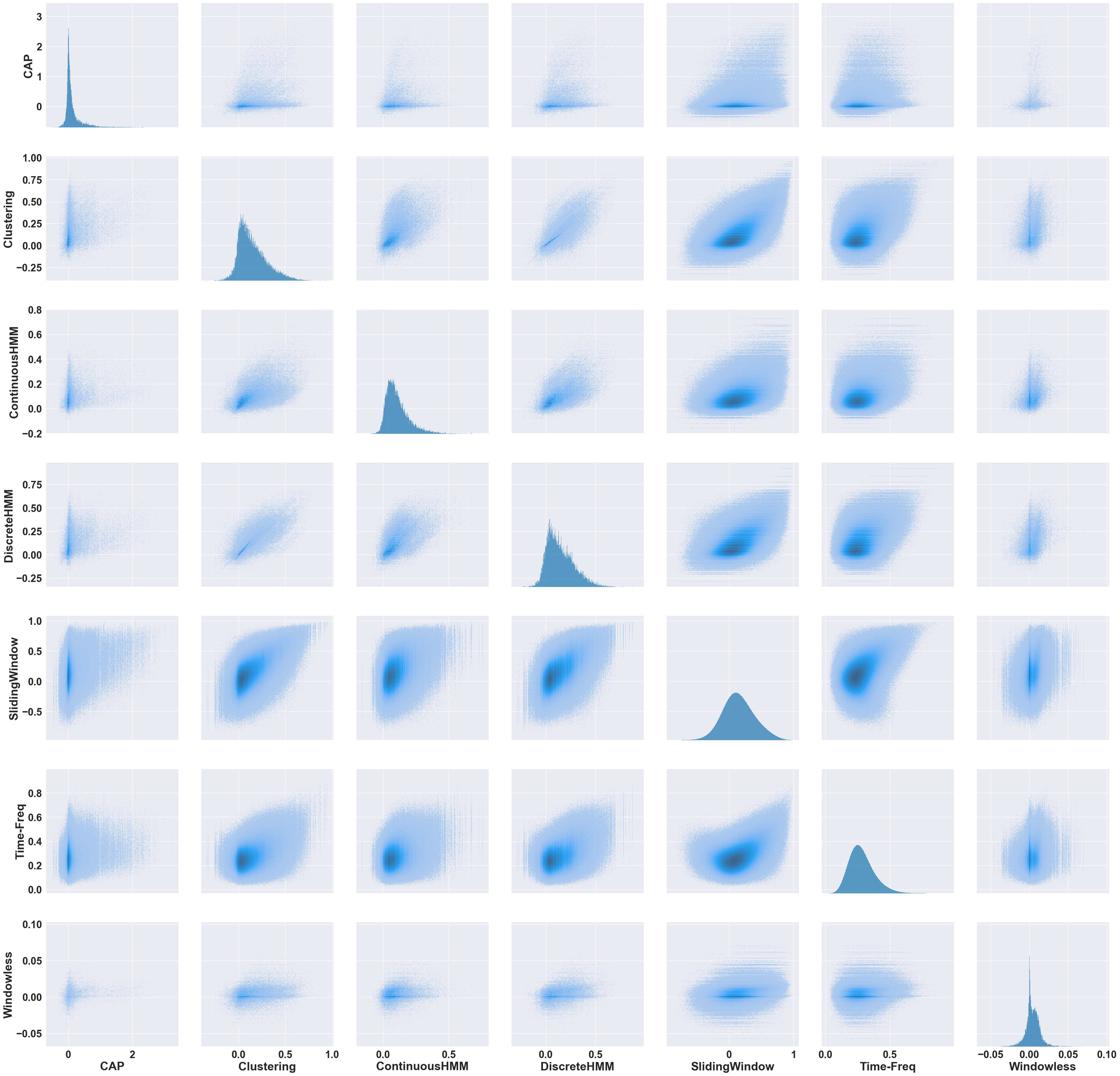

Supplement: giae009_Supplemental_Files [file giae009_supplemental_files.zip › Supplementary Files/SuppFigures/Supplementary_Fig_S15.png]

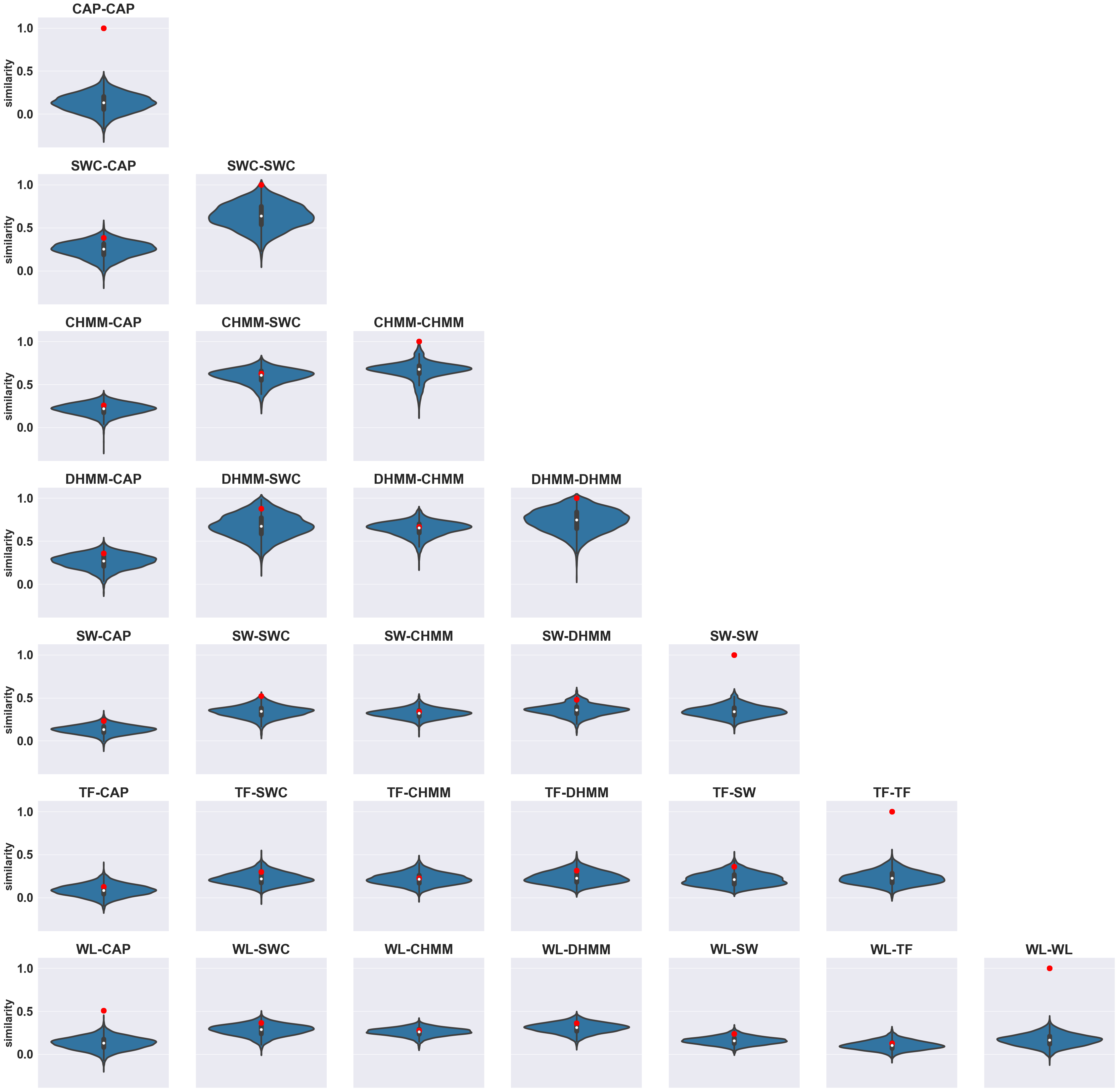

Supplement: giae009_Supplemental_Files [file giae009_supplemental_files.zip › Supplementary Files/SuppFigures/Supplementary_Fig_S29.png]

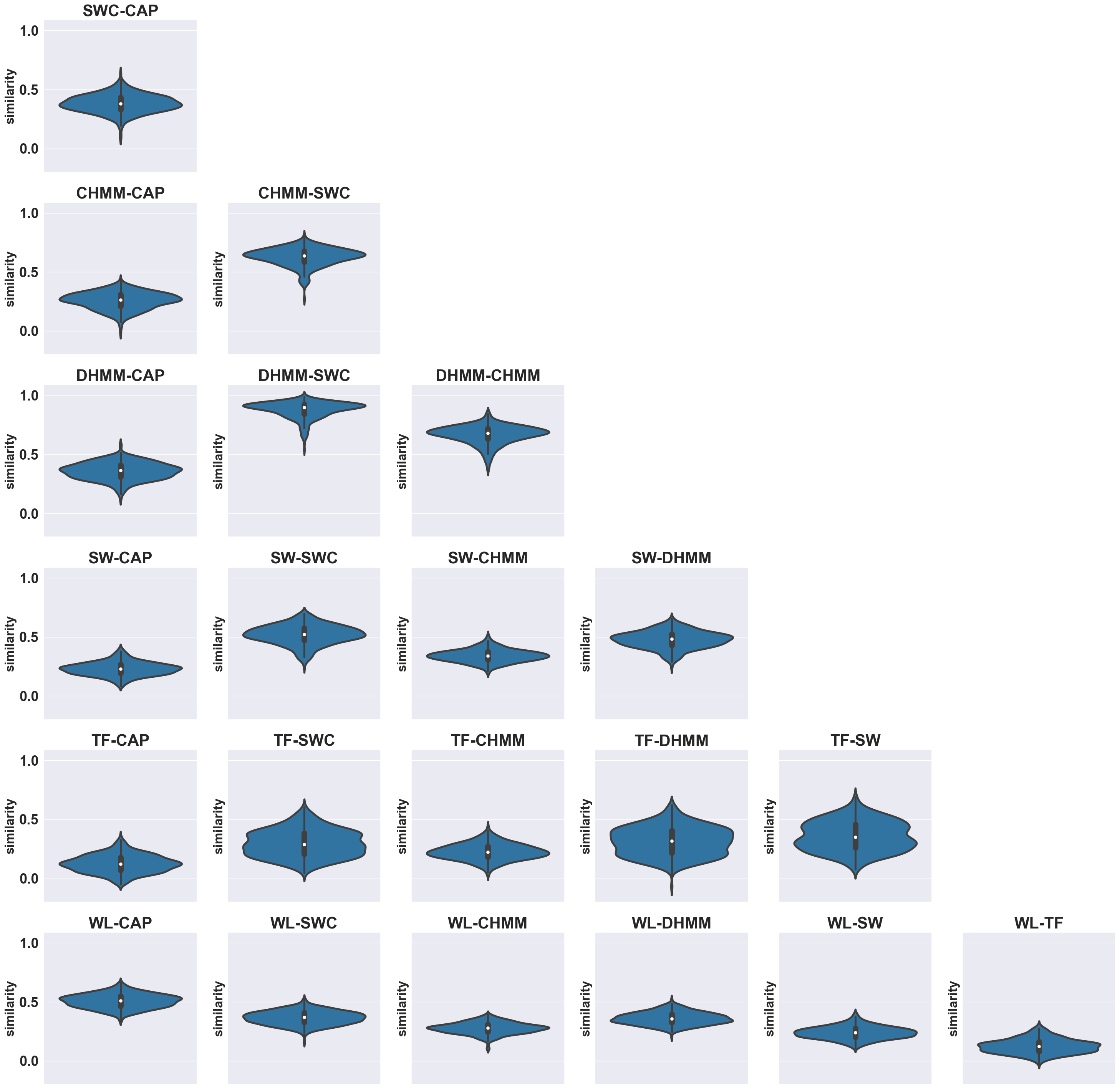

Supplement: giae009_Supplemental_Files [file giae009_supplemental_files.zip › Supplementary Files/SuppFigures/Supplementary_Fig_S17.png]

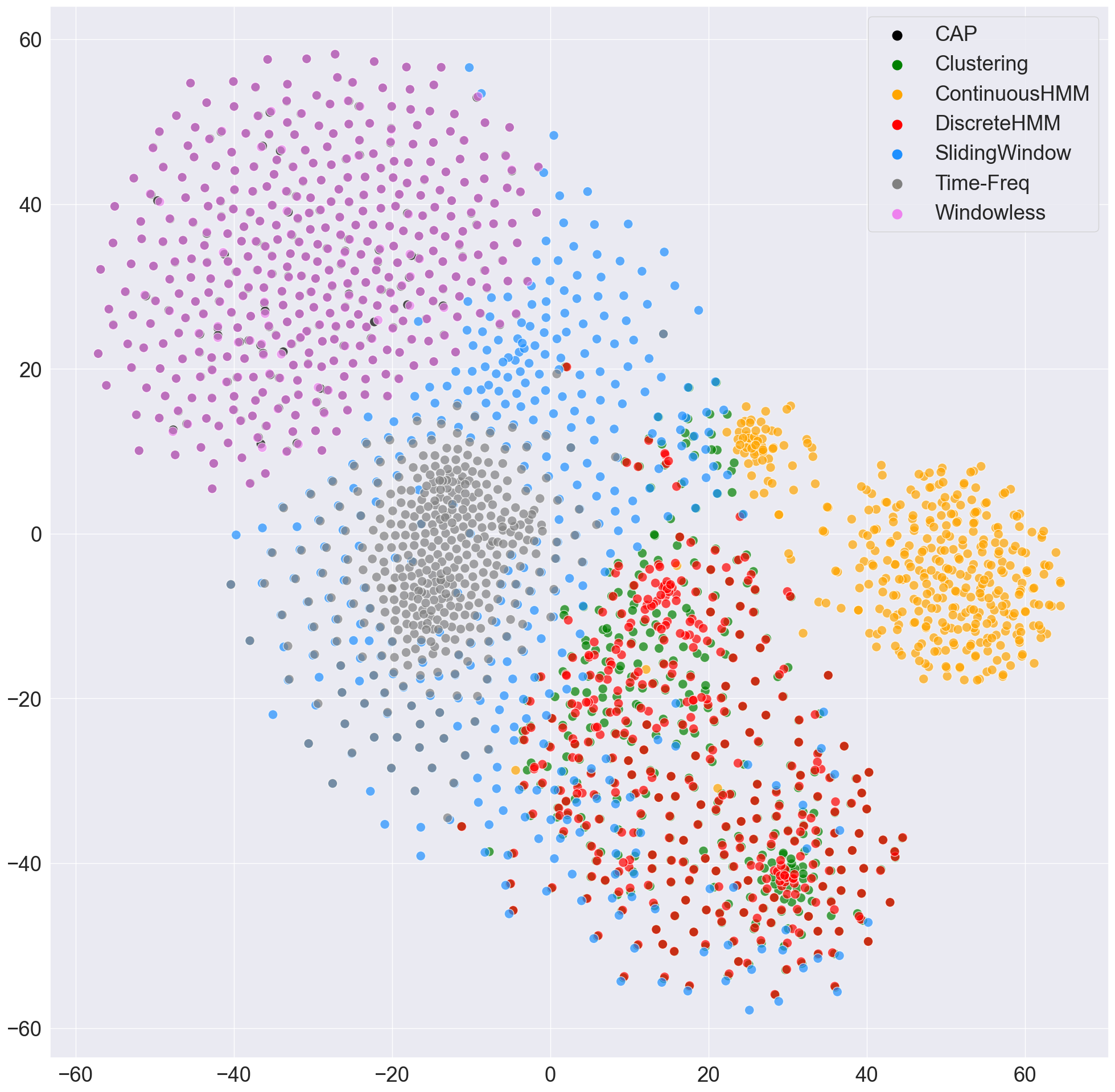

Supplement: giae009_Supplemental_Files [file giae009_supplemental_files.zip › Supplementary Files/SuppFigures/Supplementary_Fig_S16.png]

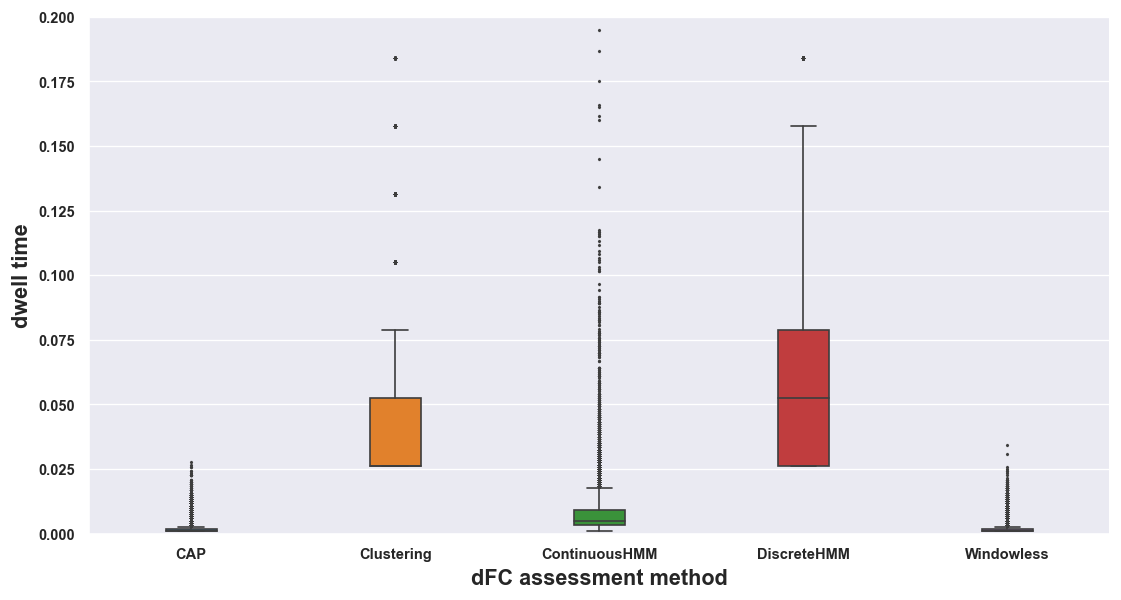

Supplement: giae009_Supplemental_Files [file giae009_supplemental_files.zip › Supplementary Files/SuppFigures/Supplementary_Fig_S12.png]

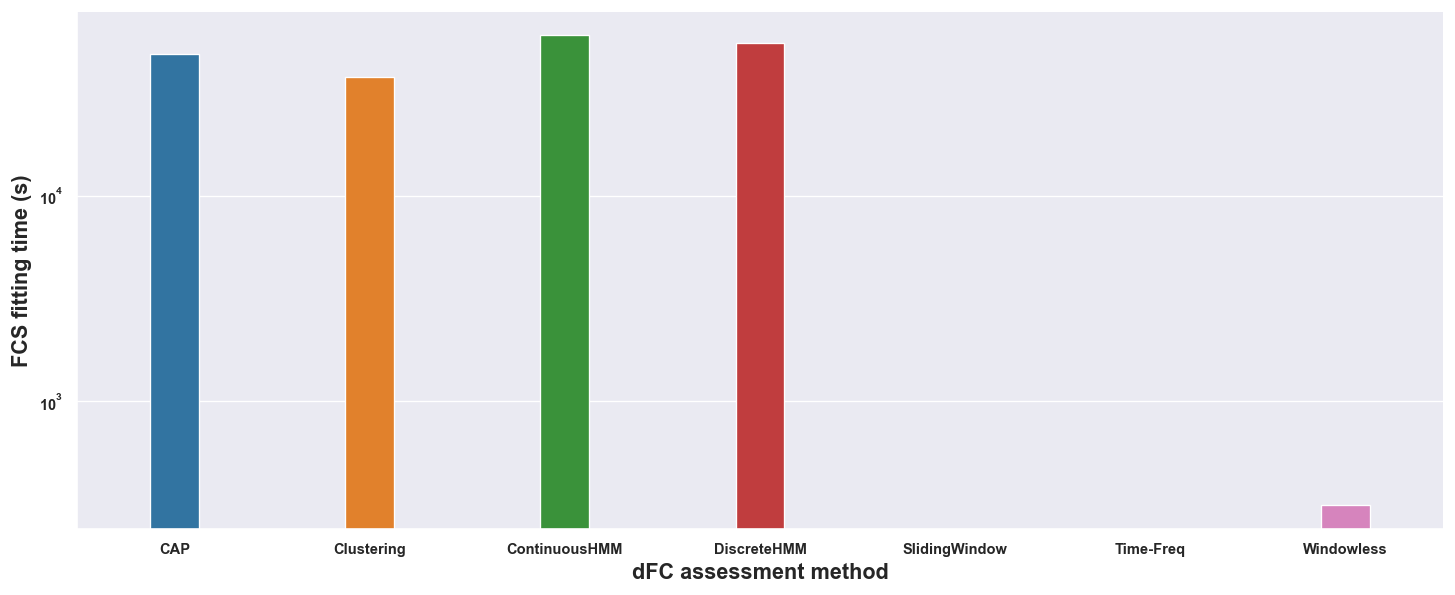

Supplement: giae009_Supplemental_Files [file giae009_supplemental_files.zip › Supplementary Files/SuppFigures/Supplementary_Fig_S13.png]

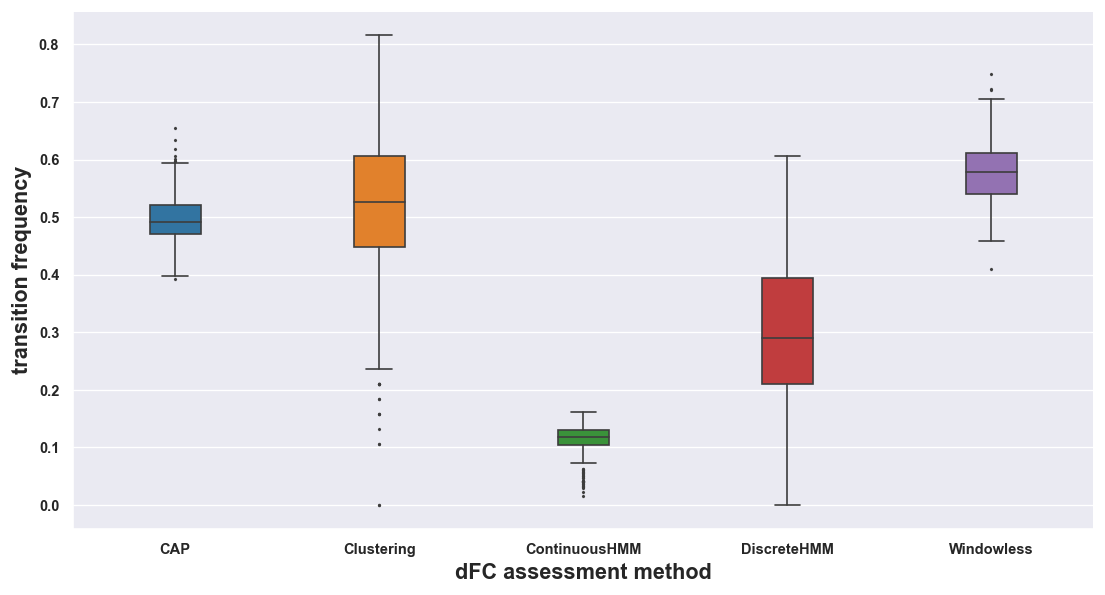

Supplement: giae009_Supplemental_Files [file giae009_supplemental_files.zip › Supplementary Files/SuppFigures/Supplementary_Fig_S11.png]

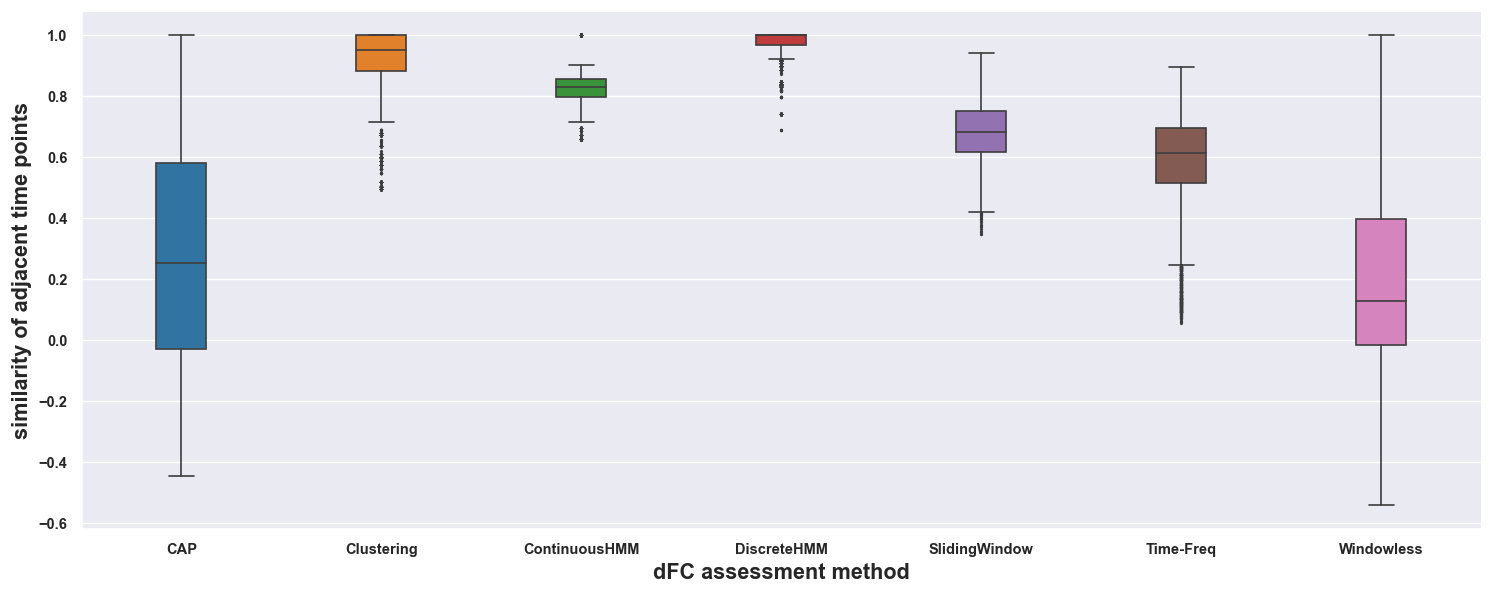

Supplement: giae009_Supplemental_Files [file giae009_supplemental_files.zip › Supplementary Files/SuppFigures/Supplementary_Fig_S10.png]
