# Supplementary material for: On the variability of dynamic functional connectivity assessment methods
Source: Gigascience. 2024 Apr 8;13:giae009. doi: 10.1093/gigascience/giae009 (PMC11000510; doi:10.1093/gigascience/giae009)
Supplement: giae009_Response_to_Reviewer_Comments_Original_Submission [file giae009_response_to_reviewer_comments_original_submission.pdf]

Author's Response To Reviewer Comments

Response to reviewer comments

Manuscript: “On the variability of dynamic functional connectivity assessment methods” (GIGA-D-23-00228)

Notes: In this document we provide responses to the reviewer comments. The responses to each comment are provided inline. Whereas the corresponding additions to the manuscript are quoted.

Editor Report:
Please register any new software application in the bio.tools and SciCrunch.org databases to receive RRID (Research Resource Identification Initiative ID) and biotoolsID identifiers, and include these in your manuscript. Computational workflows should be registered in workflowhub.eu and the DOIs cited in the relevant places in the manuscript. These will facilitate tracking, reproducibility and re-use of your tool.

Response: We have registered our toolbox in the bio.tools database (biotoolsID: biotools:pydfc) and SciCrunch.org (RRID: SCR_024729). However, registering on workflowhub.eu proved challenging, and upon careful consideration, we are inquiring whether this registration is mandatory for this article, especially considering the platform's limited usage within the neuroimaging community. We believe that the provided registrations will sufficiently enhance accessibility for readers and facilitate the reproducibility of our study, as well as the re-use of our tool. This information is also included in the manuscript (Page 9, Lines 857-867):

“PydFC is an open-source toolbox available on GitHub [55]. PydFC is registered with the SciCrunch Registry (RRID: SCR_024729) and bio.tools Registry (biotoolsID: biotools:pydfc).

Project name: PydFC
DOI: 10.5281/zenodo.10211966
Programming language: Python
Operating System(s): Platform independent
source code: https://github.com/neurodatascience/dFC
biotoolsID: biotools:pydfc
RRID: SCR_024729
License: MIT License ”

Reviewer Reports:

Reviewer #1
The authors performed an in-depth comparison of 7 dynamic functional connectivity methods. The paper includes many figures that are greatly appreciated as they clearly demonstrate the findings. Moreover, the authors developed a Python toolbox to implement these 7 methods. The results showed that the results were highly variable, although three clusters of similar methods could be detected. However, after reading the manuscript, there are some remaining questions.

Response: We appreciate the overall positive assessment of our work. We also appreciate the questions you have raised, and we have addressed them to our best capacity. Below are our responses to these questions:

R1-01: The TR and timepoints of the fMR images are shown, but other acquisition parameters such as the voxel size are missing. Could all acquisition parameters please be provided?

Response: The scan parameters were not initially provided as we utilized the Human Connectome Project

(HCP) dataset, which is publicly available. These parameters can be accessed on the HCP website. However, for the convenience of the readers, we have now incorporated these details into our manuscript (Methods, Page 3, Lines 241-253):

“Each scan consists of 1200 time points with repetition time TR=0.720 sec. The other fMRI acquisition parameters are as follows: Sequence: gradient-echo EPI; echo time TE=33.1 ms; flip angle FA=52 degrees; field of view FOV=208x180 mm (ROxPE); Matrix =104x90 (ROxPE); slice thickness =2.0 mm; 72 slices; 2.0 mm isotropic voxels; multiband factor=8; echo spacing ES=0.58 ms; bandwidth BW=2290 Hz/Px (for more details see [53]). We used the HCP FIX-denoised data, which includes additional denoising steps, i.e., de-trending, head motion correction, and denoising via spatial ICA compared to the minimally preprocessed data available in the HCP database [58,59]. Furthermore, the downloaded FIX-denoised volumetric data underwent minimal spatial smoothing with a Full Width at Half Maximum (FWHM) of 4 mm [49]. ”

R1-02: Could more information be provided on the downsampling of the 286 to 96 ROIs? How was this done and what were the 96 ROIs that were created?

Response: We have provided additional details on the downsampling process from 286 ROIs to 96 ROIs (Methods, Page 3, Lines 260-267):

“For computational reasons, we have uniformly downsampled these 286 ROIs to 96 ROIs. This downsampling process involved selecting approximately one-third of the ROIs from each RSN in a manner ensuring uniform coverage across the area of that specific RSN and an equal number of ROIs from each hemisphere (see Figure 5 in Supplementary material for a visualization of the selected 96 ROIs). Each ROI time series was z-standardized before the analysis.”

Moreover, we have added a new figure, Figure 5, to the Supplementary material (Page 18), illustrating the 96 ROIs selected for the analysis, along with a list specifying the number of ROIs selected from each RSN. We believe that this additional information enhances the clarity of our methodology.

“Figure 5. The 96 Regions Of Interest (ROIs) that were uniformly selected out of the initial set of 286 ROIs spanning across 12 Resting States Networks (RSNs). The ROIs chosen from each RSN were selected to span the entire spatial extent of the respective RSN, ensuring an equal representation from both hemispheres. The following is a breakdown of the number of ROIs selected from each RSN: Auditory (8), Cingulo-Opercular (13), Default Mode (14), Dorsal Attention (11), FrontoParietal (8), Medial-Parietal (2), Parieto-Occipital (3), SomatoMotor Hand (13), SomatoMotor Mouth (3), Salience (1), Ventral Attention (7), and Visual (13).”

R1-03: In the results it is explained that the definition of groups depended on the cutoff value of the clustering, however it is unclear how the cutoff value was determined. Could the authors elucidate how this was done?

Response: We appreciate the attention to this aspect and have provided further clarification on the determination of the cutoff value for hierarchical clustering in the manuscript. Additionally, we have added a dashed line representing the threshold value to all our hierarchical clustering figures in both the main text and the Supplementary material. The revised passage reads (Results, Pages 6-7, Lines 485-503):

“We used hierarchical clustering analysis on the pair-wise similarity matrices to investigate and summarize variation between dFC methods. The hierarchical clustering based on these average correlation matrices (Figure 3b) suggests that the methods can be classified into three groups: 1) Clustering, Continuous HMM, and Discrete HMM; 2) CAP and Window-less; 3) Sliding Window and Time-Frequency. To obtain these groups, we applied a cutoff value equal to 0.7 x the maximum distance (Ward distance) observed between different clusters (shown as a horizontal dashed line in the hierarchical clustering figures). While the definition of these groups depends on the specific clustering cutoff value used, our observations revealed pronounced intra-group similarities and comparatively lower inter-group similarities for these three

identified groups (For results obtained by other metrics see Supplementary material: Pearson correlation, Figure 25; Euclidean distance, Figure 26; and mutual information, Figure 27). Most subjects exhibited the same hierarchical clustering pattern as the one observed in the average pattern across all subjects (Figure 22 in Supplementary material)."

R1-04: The difference between the subplots in Figure 3 is a bit difficult to understand because the labels of the different methods switch places. Perhaps the same colour could be used for the cluster of the continuous HMM, Clustering and Discrete HMM method to increase readability?
Response: We appreciate the reviewer's feedback on the readability of Figure 3. While enforcing a specific order of methods in the hierarchical clustering dendrogram is not feasible due to its automatic arrangement based on distances between clusters, we have tried to enhance visual clarity. Specifically, we have now fixed the colors of the clusters associated with each of the three identified groups across all hierarchical clustering figures, both in the main text and the Supplementary material. The colors are as follows: 1) CAP, Window-less: Red; 2) Clustering, Continuous HMM, Discrete HMM: Orange; 3) Sliding Window, Time-Frequency: Green. This adjustment aims to provide a more consistent and reader-friendly representation of the identified clusters in our hierarchical clustering analyses. Please see updated version of Figure 3 on Page 16:

"Figure 3. Similarity between dFC patterns obtained from the seven examined methods as assessed by Spearman correlation: (a) The overall (combined spatial and temporal) similarity values averaged over subjects exhibit considerable variation over different method pairs for all sessions; (b)-(e): Hierarchical clustering based on (b) average overall similarity, obtained by comparing the entire dFC matrices, (c) average spatial similarity, obtained by comparing FC matrices at each time point, (d) average temporal similarity, obtained by comparing time courses of each functional connection, and (e) average inter-subject similarity, obtained by comparing inter-subject correlation values, measured by Spearman correlation. The hierarchical clustering results suggest that the methods can be grouped into three groups: 1) Clustering, Continuous HMM, and Discrete HMM: Orange; 2) CAP and Window-less: Red; 3) Sliding Window and Time-Frequency: Green. The horizontal dashed lines represent the clustering cutoff values. A slightly different pattern was obtained through temporal similarity assessment, which is due to decreased similarity of Continuous HMM and Time-Frequency with the methods in their group. The general similarity between the methods was relatively low, particularly for inter-group comparisons. For results obtained by other metrics see Supplementary material: Pearson correlation, Figure 25; Euclidean distance, Figure 26; and mutual information, Figure 27."

R1-05: Figure 4b shows that the default mode network is more variable over methods than time, while the auditory and visual are not. Could the authors explain what may underlie this discrepancy?

Response: We appreciate the insightful observation regarding Figure 4b. The variability patterns observed across different resting-state networks (RSNs) are indeed intriguing. While providing a definitive reason for this discrepancy is challenging due to the absence of a ground truth and requires further investigation through simulation studies and controlled paradigms, we have introduced a hypothesis to address this in our manuscript (Discussion, Page 9, Lines 769-786):

"Furthermore, it would shed light on the varying levels of method variability observed across different RSNs by identifying the factors that contribute to higher variability in certain RSN pairs compared to others. Our hypothesis is that the distinct Signal-to-Noise Ratios (SNRs) among the BOLD signals of different RSNs, for instance due to varying susceptibility to physiological signals, could contribute to this observation [64]. For instance, the Default Mode Network (DMN) encompasses highly vascular areas and regions that are part of the central autonomic network [65, 66] and this can lead to a different level of SNR and therefore methodological variability in DMN compared to other networks. Moreover, the pronounced time-varying connectivity patterns of the DMN, potentially arising from its role as a relay between different networks [5, 67], challenge methods aiming to accurately capture FC dynamics, thereby leading to larger methodological variability. However, a comprehensive interpretation and validation of these hypotheses require further investigation through simulation studies and controlled paradigms."

Furthermore, we also observed that intra-connections across all RSNs, including the auditory and visual networks (except for the Cingulo-Opercular network), exhibit more variability over methods than time.

R1-06: From the introduction it became clear that many studies have used dFC to study clinical populations, while I understand that no single recommendation can be given, not every clinical study might have the capacity to use all 7 methods. What would the authors recommend for these clinical studies? Would there for example be a method that would be recommended within each of the three clusters?

Response: Although this study does not intend to reveal the optimal methodologies due to the lack of a ground truth, we have added a few recommendations to the manuscript (Discussion, Page 9, Lines 826-843) based on the assumptions and individual properties of each methodology, as well as leveraging the similarity patterns observed between the methods.

"While this study does not permit unequivocal recommendations regarding an optimal methodology, given the absence of a clear ground truth, researchers may adopt a pragmatic approach by considering the assumptions and individual properties outlined for each method in alignment with their specific analytical needs. For example, given the relatively similar results produced by WL and CAP, one might favor WL over CAP due to its significantly shorter computation time (refer to Figure 17 in Supplementary material). The choice between methods such as SWC and DHMM, given their similar results, depends on the preference for implementation complexity; SWC may be preferred for simplicity, while DHMM offers a more comprehensive assessment. In cases where the dependency between time points is a critical consideration, selecting a method from the SWC-CHMM-DHMM group or the SW-TF group is advisable over a method from the WL-CAP group, while in the cases where capturing rapid transitions of FC are critical, choosing a method from the WL-CAP group may be insightful (refer to Table 1 and Figure14, Figure15, and Figure16 in Supplementary material)."

R1-07: It could be helpful if the authors create DOIs for their toolbox code bases that could be cited in a manuscript, rather than linking to bare GitHub URLs. One potentially useful guide is: https://guides.github.com/activities/citable-code/

Response: Thank you for pointing this out. It is certainly useful for our study as we aim to publish our open-source toolbox along with our paper. We have now created a DOI using Zenodo for our toolbox code on GitHub and added the DOI to the GitHub repository as well as the manuscript (Introduction, Page 3, Lines 194-209):

"To support researchers engaged in this endeavor, we implemented our framework as an open-source Python toolbox termed PydFC, which simplifies the implementation of multi-analysis dFC assessment. The toolbox enables users to apply various dFC assessment methods to their data and experiment with different hyperparameters, allowing for a comprehensive analysis of dFC variability over methods and hyperparameters. Users can compare the results of different methods within a single package, making it easier to explore and evaluate dFC assessment techniques. This toolbox is unique in providing an open-source implementation of commonly used dFC methods and could be a valuable resource for researchers seeking to explore and compare different dFC assessment techniques. The toolbox is available on GitHub [55] and is designed to be an open and collaborative package (MIT license), where contributors are welcome and the implementation of additional methods can be integrated."

Reviewer #2
This paper fills a very important gap in the literature investigating time-varying functional connectivity (or dynamic functional connectivity, dFC), by measuring analytical flexibility of seven different dFC methods. An impressive amount of work has been put up to generate a set of convincing results, that essentially show that the main object of interest of dFC, which is the temporal variability of connectivity, cannot be measured with a high consistency, as this variability is of the same order of magnitude or even higher than the changes observed across different methods on the same data. In this very controversial field, it is very remarkable to note that the authors have managed to put together a set of analysis to demonstrate this in

a very clear and transparent way. The paper is very well written, the overall approach is based on a few assumptions that make it possible to compare methods (e.g. subsampling of temporal aspects of some methods, spatial subsampling), and the provided analysis is very complete. The most important results are condensed in a few figures in the main manuscript, which is enough to convey the main messages. The supplementary materials provide an exhaustive set of additional results, which are shortly discussed one by one. Most importantly, the authors have provided an open source implementation of 7 main dfc methods. This is very welcome for the community and for reproducibility, and is of course particularly suited for this kind of contribution. A few suggestions follow.

Response: We would like to thank the reviewer for the thoughtful review and positive feedback on our work. Below are the responses to the specific points that the reviewer has raised:

R2-01: How was the uniform downsampling of 286 ROI to 96 done ? Uniform in which sense ? According to the RSN ? Were ROIs regrouped with spatial contiguity ? I understand this was done in order to reduce computational complexity and to harmonize across methods, but the manuscript would benefit from having an added sentence to explain what was done.

Response: We have not regrouped ROIs with spatial contiguity. Rather, we have retained one-third of the ROIs and removed the rest. We have now elaborated on how the downsampling from 286 ROIs to 96 ROIs was done in the manuscript. We have now provided additional details on this process in the manuscript (Methods, Page 3, Lines 260-267):

“For computational reasons, we have uniformly downsampled these 286 ROIs to 96 ROIs. This downsampling process involved selecting approximately one-third of the ROIs from each RSN in a manner ensuring uniform coverage across the area of that specific RSN and an equal number of ROIs from each hemisphere (see Figure 5 in Supplementary material for a visualization of the selected 96 ROIs). Each ROI time series was z-standardized before the analysis.”

Moreover, we have added a new figure, Figure5, to the Supplementary material (Page 18), illustrating the 96 ROIs selected for the analysis along with a list specifying the number of ROIs selected from each RSN. We believe that this additional information enhances the clarity of our methodology.

“Figure 5. The 96 Regions Of Interest (ROIs) that were uniformly selected out of the initial set of 286 ROIs spanning across 12 Resting States Networks (RSNs). The ROIs chosen from each RSN were selected to span the entire spatial extent of the respective RSN, ensuring an equal representation from both hemispheres. The following is a breakdown of the number of ROIs selected from each RSN: Auditory (8), Cingulo-Opercular (13), Default Mode (14), Dorsal Attention (11), FrontoParietal (8), Medial-Parietal (2), Parieto-Occipital (3), SomatoMotor Hand (13), SomatoMotor Mouth (3), Salience (1), Ventral Attention (7), and Visual (13).”

R2-02: Table A in figure 1 shows the important hyperparameters (HP) for each method, but the motivations regarding the choice of HP for each method is only explained in the discussion (end of page 11, "we adopted the hyperparameter values recommended by the original paper or consensus among the community for each method"). It would be better to explain it in the methods, and then only discuss why this can be a limitation, in the discussion.

Response: We appreciate the reviewer's suggestion and we have incorporated additional details regarding the choice of hyperparameter values for each method directly in the Methods section (Pages 4-5, Lines 268-418). Below are specific examples:

“Sliding Window (SW): … The main hyperparameters of this method are window length, overlap ratio, and tapered or rectangular window. The values of these hyperparameters were chosen following [5]. The overlap ratio of 0.5 was chosen to balance the temporal resolution of dFC assessment and computational demands.”

“Time-Frequency (TF): … The hyperparameters of this method include number of frequency scales, wavelet type, significance level of coherence magnitude, and considering the cone of influence or not. Following [7], a Morlet wavelet at 10 frequency scales was employed. The significance level was set to 0.95, and only the WTC indices outside the cone of influence were considered.”

“Co-Activation Patterns (CAP): … The hyperparameters of this method are the number of subject-level clusters, and the number of FC states. The number of subject-level clusters was set to 20 based on [28].”

“Sliding Window + Clustering (SWC): … The hyperparameters of this method are, in addition to the hyperparameters of the sliding window method, the number of subject-level clusters, as well as the number of FC states. The values for sliding window hyperparameters were chosen as in the sliding window method, and the number of subject-level clusters was set to 20, consistent with the CAP method.”

“Continuous Hidden Markov Model (CHMM): … The only hyperparameters of this method are the observation model type (e.g. Gaussian, or Autoregressive), and number of FC states. A Gaussian model was chosen as the observation model following [51].”

“Discrete Hidden Markov Model (DHMM): … The hyperparameters of this method include the hyperparameters of the clustering method, number of observations assumed in the observation model, and number of FC states. For the clustering method hyperparameters, the same values as the clustering method were chosen, and the number of observations to number FC states ratio, 16/24, was adopted from [28].”

R2-03: The github repository https://github.com/neurodatascience/dFC/tree/main does not reference the paper

Response: Thank you for bringing this to our attention. We have now included a reference to our paper on bioRxiv in the README file of the GitHub repository. This addition provides a clear link between the GitHub repository and the associated paper. Following the publication of our paper, we will update the link in the repository to direct to the publication for further accessibility.

R2-04: The github repository https://github.com/neurodatascience/dFC/tree/main is not documented enough. There are two very large added values in this repo : open implementation of methods, and analytical flexibility tools. The demo notebook shows how to use the analytical flexibility tools, but the methods implementation is not documented. I expect that many people will want to perform analysis using the methods as well as comparison analysis, so the documentation of individual methods should not be minimized.

Response: We appreciate your suggestion for enhanced documentation. To address this, we have included a dedicated Jupyter notebook named `dFC_methods_demo.ipynb` in our GitHub repository. This new notebook provides comprehensive guidance on how to use each of the individual dFC assessment methods implemented in the toolbox. Furthermore, we have refined our existing Jupyter notebook, `multi_analysis_demo.ipynb`, which demonstrates how to apply multiple dFC methods simultaneously on data and utilize analytical flexibility tools. We believe that these changes will significantly improve the user experience, enabling individuals to perform analyses using both the implemented methods and the analytical flexibility tools. We hope that these resources contribute to a more accessible and user-friendly experience. Thank you for this feedback and we would welcome any further suggestions or considerations.

R2-05: For the reader, it would be better to include early in the manuscript (in the introduction) the presence of the code for reproducibility. Currently, the toolbox is only introduced in the final paragraph of the discussion. It comes as a very nice surprise when reading the manuscript in full, but I think the manuscript would gain a lot of value if this paragraph was included earlier, and if the development of the toolbox was included much earlier (ie. in the abstract).

Response: This is a good point, we have incorporated this suggestion by introducing the toolbox in both the introduction and the abstract. We believe that this modification emphasizes the value of the provided toolbox for the research community. In the introduction (Page 3, Lines 194-209):

“To support researchers engaged in this endeavor, we implemented our framework as an open-source Python toolbox termed PydFC, which simplifies the implementation of multi-analysis dFC assessment. The toolbox enables users to apply various dFC assessment methods to their data and experiment with different hyperparameters, allowing for a comprehensive analysis of dFC variability over methods and hyperparameters. Users can compare the results of different methods within a single package, making it easier to explore and evaluate dFC assessment techniques. This toolbox is unique in providing an open-source implementation of commonly used dFC methods and could be a valuable resource for researchers seeking to explore and compare different dFC assessment techniques. The toolbox is available on GitHub [55] and is designed to be an open and collaborative package (MIT license), where contributors are welcome and the implementation of additional methods can be integrated.”

R2-06: We have published two papers on dFC that the authors may want to include, although these papers have investigated cerebello-cerebral dFC using whole brain + cerebellum parcellations. The first paper used continuous HMM on healthy subjects, and found correlations with impulsivity scores, while the second papers used network measures on sliding window dFC matrices on a clinical cohort (patients with alcohol use disorder). I am not sure why the authors have not found our papers in their literature, but maybe it would be good to include them. Authors need to update the final table in supplementary materials as well as the citations in the main paper.
Abdallah, M., Farrugia, N., Chirokoff, V., & Chanraud, S. (2020). Static and dynamic aspects of cerebro-cerebellar functional connectivity are associated with self-reported measures of impulsivity: A resting-state fMRI study. Network Neuroscience, 4(3), 891-909.
Abdallah, M., Zahr, N. M., Saranathan, M., Honnorat, N., Farrugia, N., Pfefferbaum, A., Sullivan, E. & Chanraud, S. (2021). Altered cerebro-cerebellar dynamic functional connectivity in alcohol use disorder: a resting-state fMRI study. The Cerebellum, 20, 823-835.
Note that in Abdallah et al. (2020), while we did not compare HMM results with other dFC methods, we did investigate the influence of HMM hyperparameters, as well as perform internal cross validation on our sample + null models of dFC.

Response: Thank you for bringing these two papers to our attention. To ensure the comprehensiveness of our work, we have included both papers in Table 2 and Table 3, as well as Figure 41 in the Supplementary material (Pages 41-43). Moreover, we have added a citation to the “Static and dynamic aspects of cerebro-cerebellar functional connectivity are associated with self-reported measures of impulsivity: A resting-state fMRI study” paper in the main text (Introduction, Page 2, Line 49), acknowledging its significance in the field of dFC research.

“... Similarly, in cognitive and behavioral applications, some studies have used sliding window and clustering methods (e.g. task prediction: [22]; cognitive and behavioral flexibility: [36]), some have used Co-Activation Patterns (CAP) analysis (e.g. naturalistic stimuli: [37]; multiple tasks: [38]), HMM (e.g. Sleep stage: [39]; Impulsivity: [40]), and very few have used multiple methods (e.g. working memory task: [41]). A complete list of the reviewed studies can be found in Tables 2, 3, and 4 in Supplementary material. …”

Minor comments:

"[..] what lies behind the methods. Instead, they reveal three groups of methods, 720 variations in dynamic functional connectivity?. " -> an extra "." was added (end of page 10).

Response: This issue was a result of a LaTeX formatting error. We have rectified the problem, and the extra "." has been removed.
