# Supplementary material for: On the variability of dynamic functional connectivity assessment methods
Source: Gigascience. 2024 Apr 8;13:giae009. doi: 10.1093/gigascience/giae009 (PMC11000510; doi:10.1093/gigascience/giae009)
Supplement: giae009_Response_to_Reviewer_Comments_Revision_1 [file giae009_response_to_reviewer_comments_revision_1.pdf]

Author's Response To Reviewer Comments

Editor Comments:
The Data Availability section needs to be updated and the GigaDB DOI cited here.
Also the GigaDB DOI needs to be added to the References list.

Response:
We have added the GigaDB DOI to the References list and updated the Data Availability section and cited the GigaDB DOI as requested (Page 9).

"The additional data supporting this work including a list of 395 subject identifiers for the subset of subjects from the HCP dataset used in this study are openly available in the GigaScience repository, GigaDB [69]."
