# Supplementary material for: On the variability of dynamic functional connectivity assessment methods
Source: Gigascience. 2024 Apr 8;13:giae009. doi: 10.1093/gigascience/giae009 (PMC11000510; doi:10.1093/gigascience/giae009)
Supplement: giae009_Reviewer_1_Report_Original_Submission — Yara Jo Toenders -- 9/15/2023 Reviewed [file giae009_reviewer_1_report_original_submission.pdf]

Reviewer Report

Title: On the variability of dynamic functional connectivity assessment methods

Version: Original Submission **Date:** 9/15/2023

Reviewer name: Yara Jo Toenders

Reviewer Comments to Author:

The authors performed an in-depth comparison of 7 dynamic functional connectivity methods. The paper includes many figures that are greatly appreciated as they clearly demonstrate the findings. Moreover, the authors developed a Python toolbox to implement these 7 methods. The results showed that the results were highly variable, although three clusters of similar methods could be detected. However, after reading the manuscript, there are some remaining questions.

- The TR and timepoints of the fMR images are shown, but other acquisition parameters such as the voxel size are missing. Could all acquisition parameters please be provided?
- Could more information be provided on the downsampling of the 286 to 96 ROIs? How was this done and what were the 96 ROIs that were created?
- In the results it is explained that the definition of groups depended on the cutoff value of the clustering, however it is unclear how the cutoff value was determined. Could the authors elucidate this how this was done?
- The difference between the subplots in Figure 3 is a bit difficult to understand because the labels of the different methods switch places. Perhaps the same colour could be used for the cluster of the continuous HMM, Clustering and Discrete HMM method to increase readability?
- Figure 4b shows that the default mode network is more variable over methods than time, while the auditory and visual are not. Could the authors explain what may underlie this discrepancy?
- From the introduction it became clear that many studies have used dFC to study clinical populations, while I understand that no single recommendation can be given, not every clinical study might have the capacity to use all 7 methods. What would the authors recommend these clinical studies? Would there for example be a method that would be recommended within each of the three clusters?
- It could be helpful if the authors create DOIs for their toolbox code bases that could be cited in a manuscript, rather than linking to bare GitHub URLs. One potentially useful guide is: https://guides.github.com/activities/citable-code/

Methods

Are the methods appropriate to the aims of the study, are they well described, and are necessary controls included? Choose an item.

Conclusions

Are the conclusions adequately supported by the data shown? Choose an item.

Reporting Standards

Does the manuscript adhere to the journal's guidelines on [minimum standards of reporting?](#) Choose an item.

Choose an item.

Statistics

Are you able to assess all statistics in the manuscript, including the appropriateness of statistical tests used? Choose an item.

Quality of Written English

Please indicate the quality of language in the manuscript: Choose an item.

Declaration of Competing Interests

Please complete a declaration of competing interests, considering the following questions:

- Have you in the past five years received reimbursements, fees, funding, or salary from an organisation that may in any way gain or lose financially from the publication of this manuscript, either now or in the future?
- Do you hold any stocks or shares in an organisation that may in any way gain or lose financially from the publication of this manuscript, either now or in the future?
- Do you hold or are you currently applying for any patents relating to the content of the manuscript?
- Have you received reimbursements, fees, funding, or salary from an organization that holds or has applied for patents relating to the content of the manuscript?
- Do you have any other financial competing interests?
- Do you have any non-financial competing interests in relation to this paper?

If you can answer no to all of the above, write 'I declare that I have no competing interests' below. If your reply is yes to any, please give details below.

I declare that I have no competing interests

I agree to the open peer review policy of the journal. I understand that my name will be included on my report to the authors and, if the manuscript is accepted for publication, my named report including any attachments I upload will be posted on the website along with the authors' responses. I agree for my report to be made available under an Open Access Creative Commons CC-BY license (http://creativecommons.org/licenses/by/4.0/). I understand that any comments which I do not wish to be included in my named report can be included as confidential comments to the editors, which will not be published.

Choose an item.

To further support our reviewers, we have joined with Publons, where you can gain additional credit to further highlight your hard work (see: https://publons.com/journal/530/gigascience). On publication of this paper, your review will be automatically added to Publons, you can then choose whether or not to claim your Publons credit. I understand this statement.

Yes Choose an item.
