# Supplementary material for: On the variability of dynamic functional connectivity assessment methods
Source: Gigascience. 2024 Apr 8;13:giae009. doi: 10.1093/gigascience/giae009 (PMC11000510; doi:10.1093/gigascience/giae009)
Supplement: giae009_Reviewer_2_Report_Original_Submission — Nicolas Farrugia -- 10/21/2023 Reviewed [file giae009_reviewer_2_report_original_submission.pdf]

Reviewer Report

Title: On the variability of dynamic functional connectivity assessment methods

Version: Original Submission **Date:** 10/21/2023

Reviewer name: Nicolas Farrugia

Reviewer Comments to Author:

Summary of review
This paper fills a very important gap in the litterature investigating time-varying functional connectivity (or dynamic functional connectivity, dFC), by measuring analytical flexibility of seven different dFC methods. An impressive amount of work has been put up to generate a set of convincing results, that essentially show that the main object of interest of dFC, which is the temporal variability of connectivity, cannot be measured with a high consistency, as this variability is of the same order of magnitude or even higher than the changes observed across different methods on the same data. In this very controversial field, it is very remarkable to note that the authors have managed to put together a set of analysis to demonstrate this in a very clear and transparent way. The paper is very well written, the overall approach is based on a few assumptions that make it possible to compare methods (e.g. subsampling of temporal aspects of some methods, spatial subsampling), and the provided analysis is very complete. The most important results are condensed in a few figures in the main manuscript, which is enough to convey the main messages. The supplementary materials provide an exhaustive set of additional results, which are shortly discussed one by one. Most importantly, the authors have provided an open source implementation of 7 main dfc methods. This is very welcome for the community and for reproductibility, and is of course particularly suited for this kind of contribution. A few suggestions follow.

Clarification questions and suggestions :

1- How was the uniform downsampling of 286 ROI to 96 done ? Uniform in which sense ? According to the RSN ? Were ROIs regrouped with spatial contiguity ? I understand this was done in order to reduce computational complexity and to harmonize across methods, but the manuscript would benefit from having an added sentence to explain what was done.

2- Table A in figure 1 shows the important hyperparameters (HP) for each method, but the motivations regarding the choice of HP for each method is only explained in the discussion (end of page 11, "we adopted the hyperparameter values recommended by the original paper or consensus among the community for each method"). It would be better to explain it in the methods, and then only discuss why this can be a limitation, in the discussion.

3- The github repository https://github.com/neurodatascience/dFC/tree/main does not reference the paper

4- The github repository https://github.com/neurodatascience/dFC/tree/main is not documented enough. There are two very large added values in this repo : open implementation of methods, and analytical flexibility tools. The demo notebook shows how to use the analytical flexibility tools, but the methods implementation is not documented. I expect that many people will want to perform analysis

using the methods as well as comparison analysis, so the documentation of individual methods should not be minimized.

5 - For the reader, it would be better to include early in the manuscript (in the introduction) the presence of the code for reproductibility. Currently, the toolbox is only introduced in the final paragraph of the discussion. It comes as a very nice suprise when reading the manuscript in full, but I think the manuscript would gain a lot of value if this paragraph was included earlier, and if the development of the toolbox was included much earlier (ie. in the abstract).

6 - We have published two papers on dFC that the authors may want to include, although these papers have investigated cerebello-cerebral dFC using whole brain + cerebellum parcellations. The first paper used continuous HMM on healthy subjects, and found correlations with impulsivity scores, while the second papers used network measures on sliding window dFC matrices on a clinical cohort (patients with alcohol use disorder). I am not sure why the authors have not found our papers in their litterature, but maybe it would be good to include them. Authors need to update the final table in supplementary materials as well as the citations in the main paper.

Abdallah, M., Farrugia, N., Chirokoff, V., & Chanraud, S. (2020). Static and dynamic aspects of cerebro-cerebellar functional connectivity are associated with self-reported measures of impulsivity: A resting-state fMRI study. Network Neuroscience, 4(3), 891-909.

Abdallah, M., Zahr, N. M., Saranathan, M., Honnorat, N., Farrugia, N., Pfefferbaum, A., Sullivan, E. & Chanraud, S. (2021). Altered cerebro-cerebellar dynamic functional connectivity in alcohol use disorder: a resting-state fMRI study. The Cerebellum, 20, 823-835.

Note that in Abdallah et al. (2020), while we did not compare HMM results with other dFC methods, we did investigate the influence of HMM hyperparameters, as well as perform internal cross validation on our sample + null models of dFC.

Minor comments

6 - "[..] what lies behind the of methods. Instead, they reveal three groups of methods, 720 variations in dynamic functional connectivity?. " -> an extra "." was added (end of page 10).

Methods

Are the methods appropriate to the aims of the study, are they well described, and are necessary controls included? Choose an item.

Conclusions

Are the conclusions adequately supported by the data shown? Choose an item.

Reporting Standards

Does the manuscript adhere to the journal’s guidelines on [minimum standards of reporting?]() Choose an item.

Choose an item.

Statistics

Are you able to assess all statistics in the manuscript, including the appropriateness of statistical tests used? Choose an item.

Quality of Written English

Please indicate the quality of language in the manuscript: Choose an item.

Declaration of Competing Interests

Please complete a declaration of competing interests, considering the following questions:

- Have you in the past five years received reimbursements, fees, funding, or salary from an organisation that may in any way gain or lose financially from the publication of this manuscript, either now or in the future?
- Do you hold any stocks or shares in an organisation that may in any way gain or lose financially from the publication of this manuscript, either now or in the future?
- Do you hold or are you currently applying for any patents relating to the content of the manuscript?
- Have you received reimbursements, fees, funding, or salary from an organization that holds or has applied for patents relating to the content of the manuscript?
- Do you have any other financial competing interests?
- Do you have any non-financial competing interests in relation to this paper?

If you can answer no to all of the above, write 'I declare that I have no competing interests' below. If your reply is yes to any, please give details below.

I declare that I have no competing interests

I agree to the open peer review policy of the journal. I understand that my name will be included on my report to the authors and, if the manuscript is accepted for publication, my named report including any attachments I upload will be posted on the website along with the authors' responses. I agree for my report to be made available under an Open Access Creative Commons CC-BY license (http://creativecommons.org/licenses/by/4.0/). I understand that any comments which I do not wish to be included in my named report can be included as confidential comments to the editors, which will not be published.

Choose an item.

To further support our reviewers, we have joined with Publons, where you can gain additional credit to further highlight your hard work (see: https://publons.com/journal/530/gigascience). On publication of this paper, your review will be automatically added to Publons, you can then choose whether or not to claim your Publons credit. I understand this statement.

Yes Choose an item.
